# Novel biosynthesis of MnO NPs using *Mycoendophyte*: industrial bioprocessing strategies and scaling-up production with its evaluation as anti-phytopathogenic agents

**DOI:** 10.1038/s41598-023-28749-z

**Published:** 2023-02-04

**Authors:** Shahira H. EL-Moslamy, I. S. Yahia, H. Y. Zahran, Elbadawy A. Kamoun

**Affiliations:** 1 Bioprocess Development Department, Genetic Engineering and Biotechnology Research Institute (GEBRI), City of Scientific Research and Technological Applications (SRTA City), New Borg El-Arab City, Alexandria 21934 Egypt; 2 Nanoscience Laboratory for Environmental and Biomedical Applications (NLEBA), Semiconductor Lab., Department of Physics, Faculty of Education, Ain Shams University, Roxy, Cairo 11757 Egypt; 3 Polymeric Materials Research Department, Advanced Technology and New Materials Research Institute (ATNMRI), City of Scientific Research and Technological Applications (SRTA-City), New Borg Al-Arab City, Alexandria 21934 Egypt; 4 Nanotechnology Research Center (NTRC), The British University in Egypt (BUE), El-Sherouk City, Cairo 11837 Egypt; 5 Laboratory of Nano-Smart Materials for Science and Technology (LNSMST), Department of Physics, Faculty of Science, King Khalid University, Abha 9004, Saudi Arabia

**Keywords:** Biological techniques, Biotechnology, Microbiology, Molecular biology, Plant sciences, Nanoscience and technology

## Abstract

This report provides the first description of the myco-synthesis of rod-shaped MnO NPs with an average crystallite size of ~ 35 nm, employing extracellular bioactive metabolites of endophytic *Trichoderma* *virens* strain EG92 as capping/reducing agents and MnCl_2_·4H_2_O as a parent component. The wheat bran medium was chosen to grow endophytic strain EG92, which produced a variety of bioactive metabolites in extracellular fraction, which increases the yield of MnO NPs to 9.53 g/l. The whole medium and fungal growth conditions that influenced biomass generation were optimized as successive statistical optimization approaches (Plackett–Burman and Box–Behnken designs). The production improvements were achieved at pH 5.5, WBE (35%), and inoculum size (10%), which increased X_max_ to twelve-folds (89.63 g/l); thereby, P_max_ increased to eight-folds (82.93 g/l). After 162 h, X_max_ (145.63 g/l) and P_max_ (99.52 g/l) on the side of µ_max_ and Y_X/S_ were determined as 0.084 and 7.65, respectively. *Via* *Taguchi* experimental design, fungus-fabricated MnO NPs reaction was improved by adding 0.25 M of MnCl_2_·4H_2_O to 100% of fungal extract (reducing/capping agents) and adjusting the reaction pH adjusted to ~ 5. This reaction was incubated at 60 °C for 5 h before adding 20% fungal extract (stabilizing agent). Also, P_max_ was raised 40-fold (395.36 g/l) over the BC. Our myco-synthesized MnO NPs exhibit faster and more precise antagonistic actions against phytopathogenic bacteria than fungi; they could be employed as an alternative and promised nano-bio-pesticide to manage a variety of different types of disease-pathogens in the future.

## Introduction

Nowadays, the application of nanotechnology is exponentially increasing in different therapeutic and agricultural activities, such as antibiotics, anticancer, antimicrobial agents, and bio-fertilizers^1^. One of the challenges in modern nanotechnology is the development of reliable, safe protocols for the synthesis of nanoparticles. Different physical and chemical techniques have been seen in synthesizing so many nanoparticles and nanostructures. Recently, these methodologies were reported as dangerous and expensive methods due to dependence on unsafe substances that could cause potential risks to the ecosystem^2^. Therefore, exploring innovative, cost-effective, non-toxic, and eco-friendly sustainable approaches should be of critical interest. So, green nanotechnology has suggested developing cost-effective and environmentally sustainable techniques to fabricate metallic nanoparticles.

There are many metals and metal oxides nanoparticles such as Ag, Au, Fe_2_O_3_, CaO, MgO, TiO_2_, ZnO, and CuO nanoparticles that different biological sources have biosynthesized^3–7^. Fungi, bacteria, plants, and even algae can be used effectively as a green factory for fabricating nanoparticles that hold immense potential applications, especially in the biomedical and agricultural sectors^8–10^. Therefore, the green synthesis of nanoparticles can be controlled by the produced bioactive metabolites such as protein, amino acids, carbohydrates, alkaloids, phenols, and even enzymes that are used as reductant and stabilizing agents. But various biological sources could not be applied in agriculture sectors due to their pathogenic properties. So it’s highly plausible that using safe bioactive molecules that could be extracted from safe biological cells^2,11^. There are several rhizosphere-isolated microorganisms (soil microbes) that have been largely exposed as nanoparticles synthesizers few reports on endophytes (plant tissues microbes). So, it is important to focus on these promising biological routes of nano-factories that produced different metallic nanoparticles with attractive sizes and shapes for different biological applications, including antimicrobial, cytotoxic, and antioxidant properties^12,13^. Microorganisms such as bacteria, actinomycetes, and fungi that vivid within plant tissues or cells without triggering any injury to their host have been called endophytes. They considered attractive candidates to open up a new line of professional biosynthesis systems linked to biology and nanotechnology. This approach is cost-effective, environmentally sustainable, and can provide nanoparticles with a better-specified size and shape without many metallic ions^14,15^. Fungal endophytes (mycoendophytes), especially *Trichoderma* spp., are producing a wide variety of novel safe bioactive metabolites (flavonoids, alkaloids, polysaccharides, and enzymes) that could be used as a potential source for producing nanoparticles by intracellular and extracellular methods. Because secreted intra and extracellular bioactive molecules play the main role in the biosynthesis of nanoparticles (bottom-up approach) by reducing metal salts, then metal ions. The fungal-mediated eco-friendly fabrication method of nanoparticles has many advantages, such as the simplicity with which the fermentation process can be scaled up, downstream processing, besides the cost-effectiveness of a biomass production line, and the potential to fabricate nanoparticles efficiently. Therefore, a variety of filamentous fungi have effectively been applied for extracellular and intracellular biosynthesis of different metals, and metal oxides nanoparticles^3,16^.

Several reports are interested in bio-synthesis or (green synthesis) of MnO NPs using plant and bacterial extracts; however, no study reports the biosynthesis of MnO NPs using the extracted bioactive metabolites from fungi especially mycoendophytic strains. Herein, the application of fungal cells as a green producer candidate for bio-fabrication of nanoparticles has been gained significant interest in the nanobiotechnology community. Also, for industrial scaling-up biosynthesis of nanoparticles, various physicochemical parameters, besides all microbial culturing conditions, were optimized to produce homogeneous MnO NPs, with satisfied antimicrobial activity. Thus, all the above aims were successfully studied statistically to get the industrial production line for MnO NPs using one of the proficient endophytes.

## Results and discussion

According to application fields, nanotechnology involves various approaches for nanostructures materials such as physical, chemical, and biological techniques. Since the biological fabrication method was emerged as a promising eco-friendly technique to overcome the side effects accompanied by physical and chemical fabrication methods^8^. So, the new branch of nanotechnology was appeared recently entitled nano-biotechnology, where nanoparticles were fabricated biologically using extracted bioactive metabolites from biological entities such as plants, algae, bacteria, and fungi^1,9,10,17^. This biological method is considered a commercially viable, clean, eco-friendly, and non-toxic technique to fabricate some nanoparticles applied in medicine and agriculture^12,18^. For scaling up fabricated nanoparticles, a new expression that appeared in nanotechnology was called “industrial nanotechnology.” Nowadays, large-scale fabrication of nanoparticles is an obligatory requirement for developing several industries without destroying human health and the environment using toxic substances or high energy^15,19^. So, the new promising scientific field appeared termed industrial nano-biotechnology. The cost-effective biological-process was applied to reduce the health risks issues and environmental downsides as reported in the present work.

### Antimicrobial activity of biosynthesized MnO NPs

**Figure 1:**
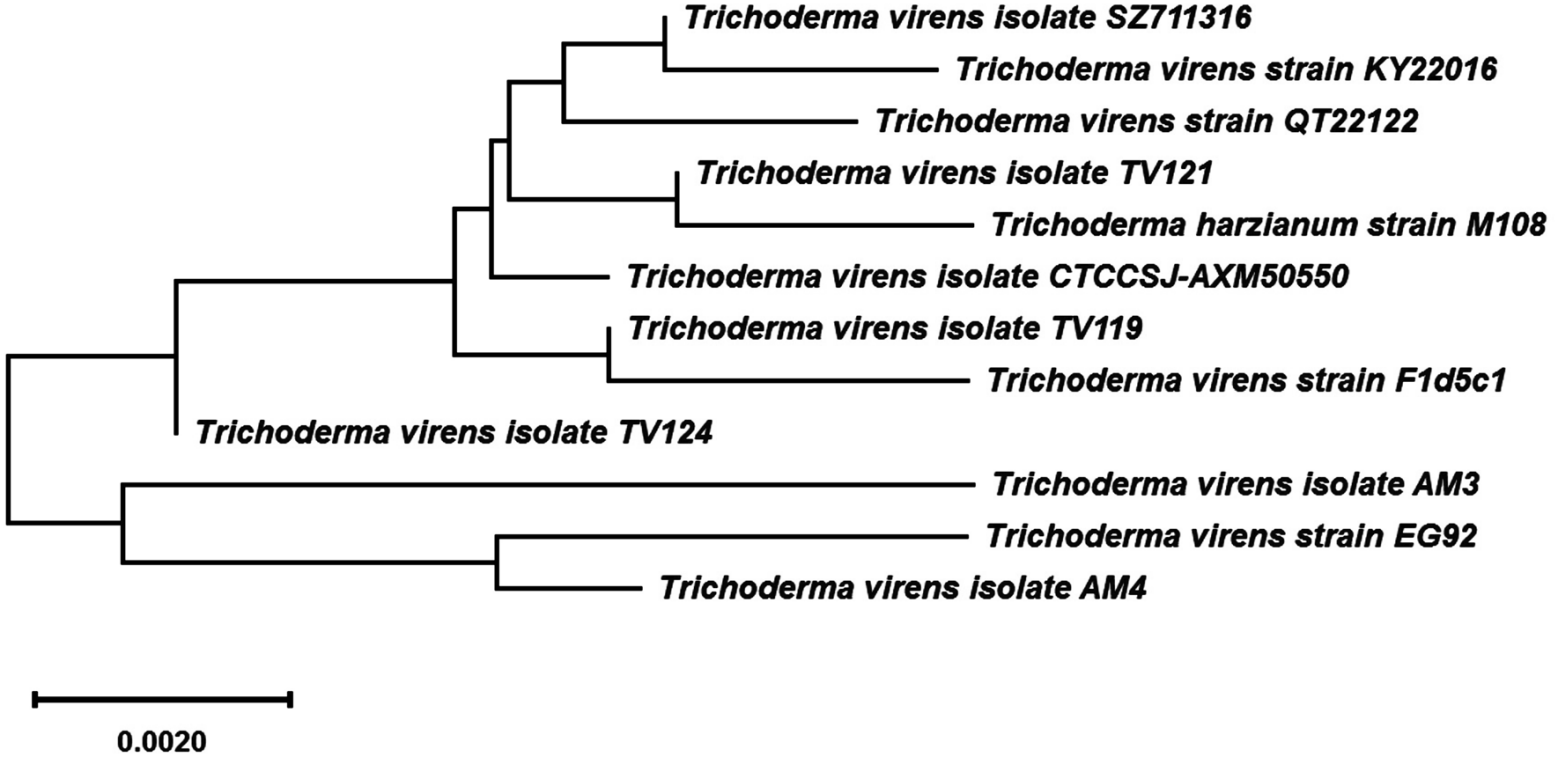
Phylogenetic tree and diversity of partial sequence of small and large subunit ribosomal RNA genes (internal transcribed spacer 1, 5.8S ribosomal RNA gene, and internal transcribed spacer 2) in endophytic *Trichoderma* *virens* strain EG92 (MF429775) compared with reference strains. This tree constructed using the neighbour-joining and maximum likelihood analysis (*MEGA10.1.7* *software* *2020*). The sequence divergence is indicated by a scale bar.

In recent times, fungal cells were discovered as potent bio-factories for the fabrication of some nanoparticles because they have various bioactive metabolites. Since most fungi require a small number of nutrients, easy to handle, and can be filtered, the fungi are preferred over other biological cells due to the saving of considerable investment costs for the fabrication of nanoparticles^3^. Mycoendophyte (endosymbiont) inhibits plant tissues without destroying the host cells. During the last decades, these mycoendophytes have been of great attention due to their produced unlimited bioactive metabolites, such as phenol, steroids, flavonoids, peptides, etc^20–22^. These mycoendophytes are relatively unexplored as a potential source of mixed bioactive metabolites that are used for biosynthesis of nanoparticles^1,8^. While, a promising antimicrobial agent might be organic metabolites; for example, enzymes or inorganic compounds as metals depending on their wide-spectrum antimicrobial activities and toleration of industrial large-scale processing conditions^5^. Recently, metal oxide nanoparticles explored as a potential class of antimicrobial agents due to their potential applications in food safety depending on their shape, size, stability, and surface properties^16,23,24^. Particularly, MnO NPs have significant distinctive physical, chemical, electrical, magnetic, and catalytic properties, which concerned extensively researches interests. Most of the scientific reports focused on estimating chemical, physical, and catalytic properties besides the application of MnO NPs using its electronic properties and catalytic activities; however, its antimicrobial activities were investigated rarely^24^. Several reports are interested in biosynthesis or (green synthesis) of MnO NPs using plant and bacterial extracts; however, no study reports the biosynthesis of MnO NPs using the extracted bioactive metabolites from fungi, especially mycoendophytic strains^25^. So, exploring myco-synthesized MnO NPs using safe, cheap, and the simplest potential bio-factories such as *Trichoderma* sp., possess novel antimicrobial activities considered a proficient modern application in many fields, especially in the agriculture sectors. Herein, different mycoendophytic isolates were used to synthesize MnO NPs, since the final fabricated reaction was recognized easily via its color changed from yellow to reddish-brown. Subsequently, the fabricated MnO NPs were tested as an antimicrobial agent against phytopathogens then the proficient isolate that produced the most effective MnO NPs as an antimicrobial agent was selected. Since the most promising antibacterial activities (*P* ≤ 0.05) were recorded against phytopathogenic bacteria such as *Erwinia* *amylovora* (34.0 ± 2.69 mm) followed by, *Acetobacter* *pasteurianus* (30.0 ± 2.45 mm), and *Erwinia* *persicina* (28.5 ± 5.4 mm). Besides, the mycosynthesized MnO NPs were tested as an antifungal agent, and the highest antifungal activities were measured against *Aspergillus* *flavus* (24.5 ± 3.20 mm), and *Aspergillus* *niger* (20.5 ± 2.06 mm), followed by *Fusarium* *solani* (19.25 ± 3.26 mm) as shown in Table 1. As a result, the chosen isolate was genetically identified using the given rDNA sequence data using GenBank Blast analysis. This isolate was submitted in the GenBank database as endophytic *Trichoderma* *virens* strain EG92 with the accession number MF429775 because it was completely similar to *Trichoderma* *virens*, as seen in Fig. 1.

**Table 1:** Antimicrobial efficacy of the biogenic synthesized MnO NPs using some phytopathogenic fungi and bacteria.

		Zone of inhibition (mm)			Zone of inhibition (mm)
Phytopathogenic fungi	*Fusarium* *solani*	19.25 ± 3.26	Phytopathogenic bacteria	*Clavibacter* *michiganensis*	22.5 ± 1.8
	*Fusarium* *moniliforme*	17.0 ± 3.5		*Erwinia* *carotovora*	24.75 ± 2.68
	*Helminthosporium* sp.	13.0 ± 2.54		*Acetobacter* *pasteurianus*	30.0 ± 2.45
	*Alternaria* *alternate*	10.25 ± 3.03		*Erwinia* *amylovora*	34.0 ± 2.69
	*Aspergillus* *flavus*	24.5 ± 3.20		*Erwinia* *persicina*	28.5 ± 5.4
	*Aspergillus* *niger*	20.5 ± 2.06			
	*Phytophthora* *arenaria*	16.0 ± 2.92

### Characterization of myco-synthesized MnO NPs

**Figure 2:**
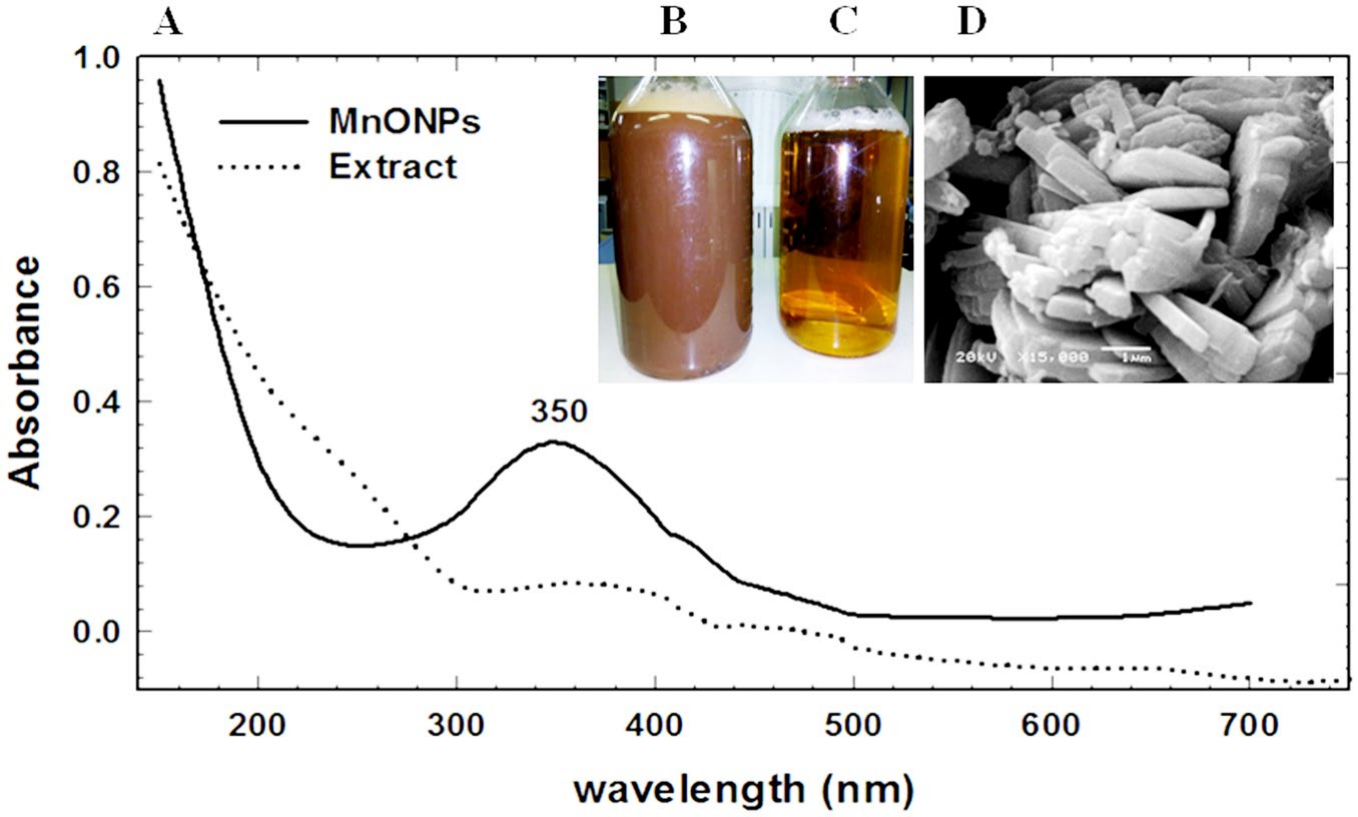
(**A**) UV–Vis spectra of fungus-fabricated MnO NPs shown surface plasmon resonance peak at 350 nm, (**B**) The final fabricated MnO NPs, (**C**) The extract of endophytic *Trichoderma* *virens* strain EG92, and (**D**) SEM image of fungus-fabricated MnO NPs shows its rod-shape structure.

In this study, the myco-synthesized MnO NPs were first detected by the naked eye when the reaction color changed from yellow to yellowish-brown and reddish-brown suspension, as presented in Fig. 2B,C; due to the excitation of the surface plasma vibrations^26^. Additionally, UV–Vis absorption intensity peaks get increased with maximum absorbance at 350 nm, which confirms the mycosynthesis of MnO NPs as shown in Fig. 2A. So, the reaction color and UV–Vis spectrum indicate the mycosynthesis of well reduced/stabilized MnO NPs was completed successfully. The UV–Vis spectroscopy is the most widely used technique to examine the optical properties of produced nanoparticles by detecting the broad absorption peak^18,27^. According to the previous reports, the UV–Visible absorption spectra of MnO NPs were ranged from 284 to 400 nm because of *n* → π⃰ and π → π⃰ transitions^11,24,25^. As well, different absorption peaks of MnO NPs indicated a difference in shape and size variations of fabricated nanoparticles according to the synthesis method^1,10^.

**Figure 3:**
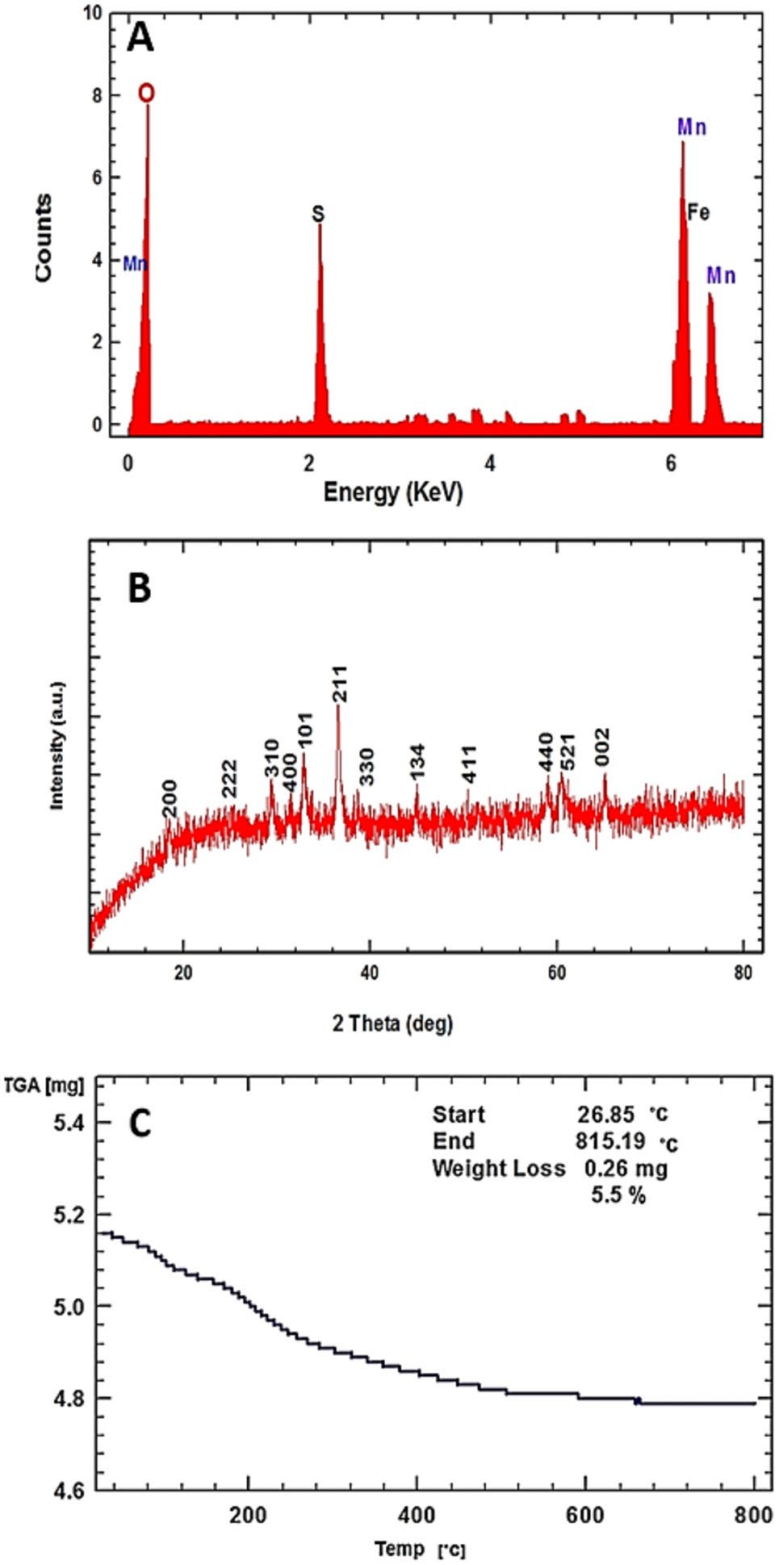
Characterization of fungus-fabricated MnO NPs using endophytic *Trichoderma* *virens* strain EG92 extract via EDX analysis (**A**), TGA thermal profile (**B**), and XRD analysis (**C**).

Previously, different shapes and sizes of synthesized nanoparticles depended on reducing/capping agents^6,25^. So, SEM was used to analyze the morphological characters of our myco-synthesized MnO NPs. The morphological character of fabricated MnO NPs was detected by SEM investigation (Fig. 2D), which exhibits the occurred agglomeration during the biosynthesis reaction. SEM image shows a very clear formed MnO NPs at the rod-shaped morphology. Besides, its elemental composition was determined via EDX analysis, which shows a strong and several signals of Mn along with a signal of O, which might have originated from the bioactive metabolites (capping organic materials) (Fig. 3A). Also, the EDX profile shows the myco-synthesized-MnO NPs as a high-purity content composed of Mn (64.45%) and O (35.5%).

Figure 3B shows the TGA thermo-profile of myco-synthesized MnO NPs, that tested at different temperatures up to 800 °C. The resulting slope of the TGA curve shows a small weight loss of about 5.5% observed at 815 °C, which might be due to the release of water, un-reacted and coated bioactive metabolites that surrounded the surface of myco-synthesized MnO NPs. Also, the XRD pattern of myco-synthesized MnO NPs using endophytic *Trichoderma* *virens* strain EG92 is completely closed to the standard pattern of body-centered tetragonal α-MnO (JCPDS file No.44-0141), that confirmed the production of MnO NPs is successfully accomplished (Fig. 3C). The sharp diffraction peaks were detected at *2θ* of 18.56°, 28.90°, 37.34°, and 63.74°, that indexed into (200), (310), (211), and (512) facets, respectively. Via XRD patterns, the average crystallite size for myco-synthesized MnO NPs is calculated using the Scherrer’s formula as 35 nm.

**Figure 4:**
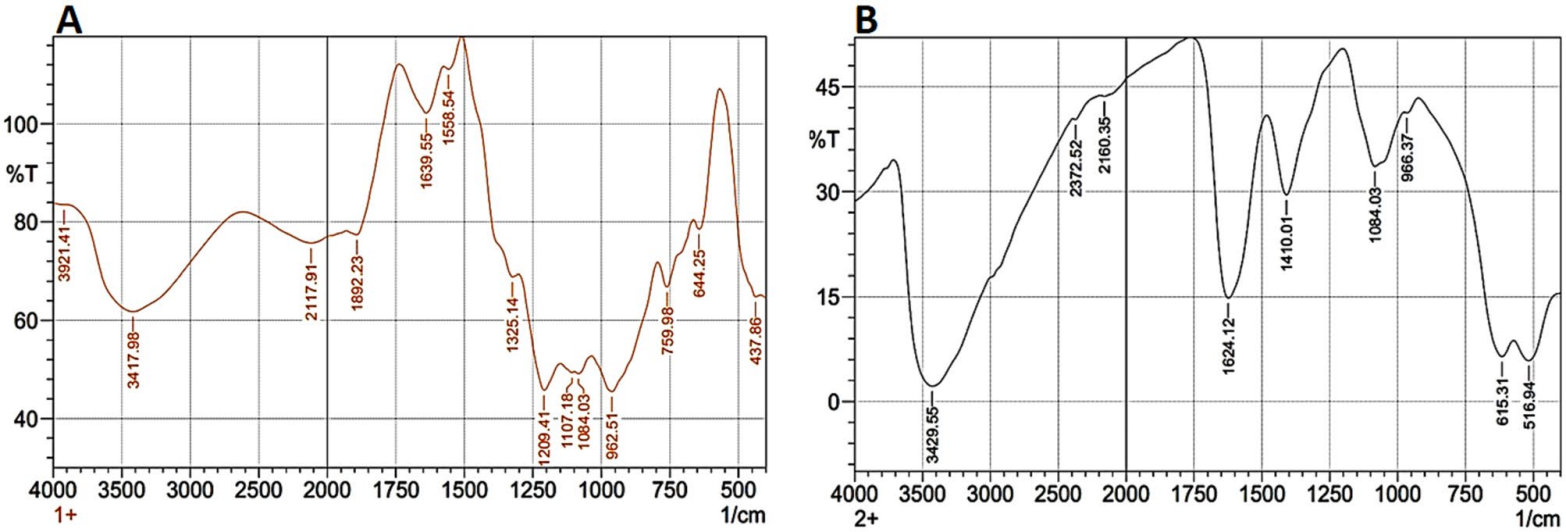
FTIR spectra of the extract of endophytic *Trichoderma* *virens* strain EG92 (**A**), and the fungus-fabricated MnO NPs (**B**).

All endophytes produced different secondary metabolites (flavonoids, terpenoids, sugars, ketones, aldehydes, carboxylic acids, etc.) to protect their hosts against predators and stress conditions^25,28^. These bioactive metabolites might be responsible for the reduction of nanoparticles^18,29^. So, the extracted phytochemicals from tested endophytic *Trichoderma* *virens* strain EG92 were analyzed. FTIR spectroscopy was used to identify the bioactive metabolites in the fungal extract that participate as reducing-capping and stabilizing agents into fabrication reaction of MnO NPs; as shown in Fig. 4A. Since the possibility of biomolecules responsible for capping/stabilizing of myco-synthesized MnO NPs; especially low content of inorganic and organic matters which are detected via FTIR analysis. The sharp absorption peaks were observed at ν 3921 cm^−1^ (O–H stretching vibration), ν 2117 cm^−1^ (C–C stretching vibration), ν 1639 cm^−1^ (C = stretching vibration), ν 1107 cm^−1^ (CH_3_ deformation vibration), ν 1064 cm^−1^ (amine group), ν 962 cm^−1^ (an aromatic group), ν 759 cm^−1^ (CH_2_ deformation vibration), ν 644 cm^−1^ (–OH group), and ν 437 cm^−1^ (C–C skeleton vibration). These indicate different functional groups might be involved in myco-synthesized MnO NPs.

In Fig. 4B, different FTIR bands disappeared into biosynthesized spectrums, such as ν 1325, 1209, and 1107 cm^−1^ assigned to the vibrations of O–H, O–H–O, and CH_3_ groups. Besides, the band has appeared at ν 1410 cm^−1^, which indicates the presence of C–H deformation vibration. So, these changes in the FTIR spectrum could be recognized as the active groups used to complete the biosynthesis of MnO NPs. While, meta-oxygen Mn–O–Mn bending vibrations of myco-synthesized MnO NPs were also identified in bands below ν 750 cm^−1^, recorded at ν 615 and 516 cm^−1^. The presence of phenols, alkaloids, carbohydrates, amino acids, and protein biomolecules in fungal extract and myco-synthesized MnO NPs might assist in converting Mn-ions into MnO NPs and coat them with a shield of various biomolecules, according to the observed peaks and phytochemical analysis results.

### Comparative evaluation study for the cultivation of endophytic *TrichodermaVirens* strain EG92

**Figure 5:**
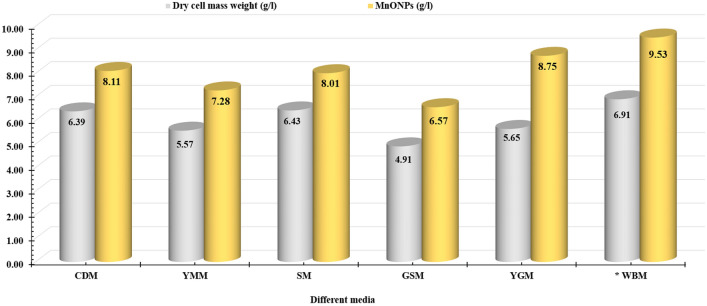
Different media that selected for cultivation of endophytic *Trichoderma* *virens* strain EG92 to fabricate MnO NPs. Czapek-Dox medium (CDM), Yeast Malt medium (YMM), Synthetic medium(SM), Glucose Soyabean meal medium (GSM), Yeast glucose medium (YGM), and Wheat bran medium (WBM).

Fungal cells normally produce important metabolites that convert toxic matters into non-toxic matters. These metabolites might be responsible for the bio-fabrication of nanoparticles since some bioactive molecules play a key role in this method^30–32^. Generally, fungal cells were used to synthesize nanoparticles extracellularly and intracellularly, especially endophytic fungi that secreted many bioactive metabolites such as proteins and phytochemicals polypeptides, and enzymes outside the hyphae and others were trapped into cells^33^. As compared to other microbes, endophytic fungi have greater tolerance and binding capacity of metal salts; hence these fungal cells have an advantage for large-scale production of nanoparticles, including efficient, low-cost downstream processes. Besides, further reports revealed that endophytic fungi have slower kinetics, so the shape and size of produced nanoparticles have long-time stability, especially produced extracellularly^31^. Here, the biosynthesis reaction of MnO NPs via the endophytic *Trichoderma* *virens* strain EG92 depended on its produced bioactive metabolites used as reducing/capping and stabilizing agents. The cultivation of endophytic *Trichoderma* *virens* strain EG92 was studied using different industrial fungal media to maximize its production of bioactive metabolites, as shown in Fig. 5.

The endophytic *Trichoderma* *virens* strain EG92 was grown well using all tested media (CDM, YMM, SM, GSM, YGM, and WBM). However, the proficient medium was WBM, which produced the highest cell mass weight (6.91 g/l) and MnO NPs yield (9.53 g/l), selected for further studies. Subsequently, the produced bioactive metabolites were distinguished into fungal cytoplasmic and extracellular fractions to detect the proficient cellular compartment used to maximize the MnO NPs mass weight (Table 2).

**Table 2:** Screening study to detect the produced phytochemical metabolites into endophytic *Trichoderma* *virens* strain EG92 fractions which cultivated using Wheat bran medium.

Bioactive metabolites	Fungal fractions	
	* Extracellular	Cytoplasmic
Alkaloids	+ ve	+ ve
Flavonoids	−ve	−ve
Tannins	+ ve	−ve
Phenols	+ ve	+ ve
Steroids	+ ve	−ve
Saponins	−ve	−ve
Terpenoids	+ ve	−ve
Total carbohydrate (μg/ml)	21.65	14.69
Total protein (mg/µl)	16.3	12.65

*Denotes the effective cellular compartment (extracellular fraction) that was used to maximise the mass weight of the MnO NPs.

The cultivated endophytic strain EG92 using WBM produced alkaloids and phenols, and low quantities of total carbohydrates (14.69 mg/µl), and total protein (12.65 mg/µl) into a cytoplasmic fraction that used to fabricate MnO NPs (2.03 g/l). Whereas the tested extracellular fraction has different bioactive metabolites such as alkaloids, tannins, phenols, steroids, and terpenoids, as well as quantities of total carbohydrate (21.65 μg/ml) and total protein (16.3 mg/µl) that produced 9.53 g/l of MnO NPs. Finally, WBM was chosen to cultivate the endophytic strain EG92 that produced different bioactive metabolites into extracellular fractions, maximizing MnO NPs yield to 9.53 g/l. The cultivation of Trichoderma strains using wheat bran extract was strongly supported by former reports^34,35^. Since organic matter extracted from agricultural waste was generally used as a non-hazardous, cost-effective, rich source of nutrients to cultivate microbial cells, especially fungal cells. Wheat bran extracts showed a lot of available proteins, amino acids, elements, and carbohydrates, so it has previously been reported as a qualified growth medium for low-cost cultivation of *Trichoderma* spp. using solid-state fermentation; however it has only been used rarely in a submerged fermentation system^36^.

### Bioprocessing strategy for optimization of endophytic strain EG92 biomass production

**Figure 6:**
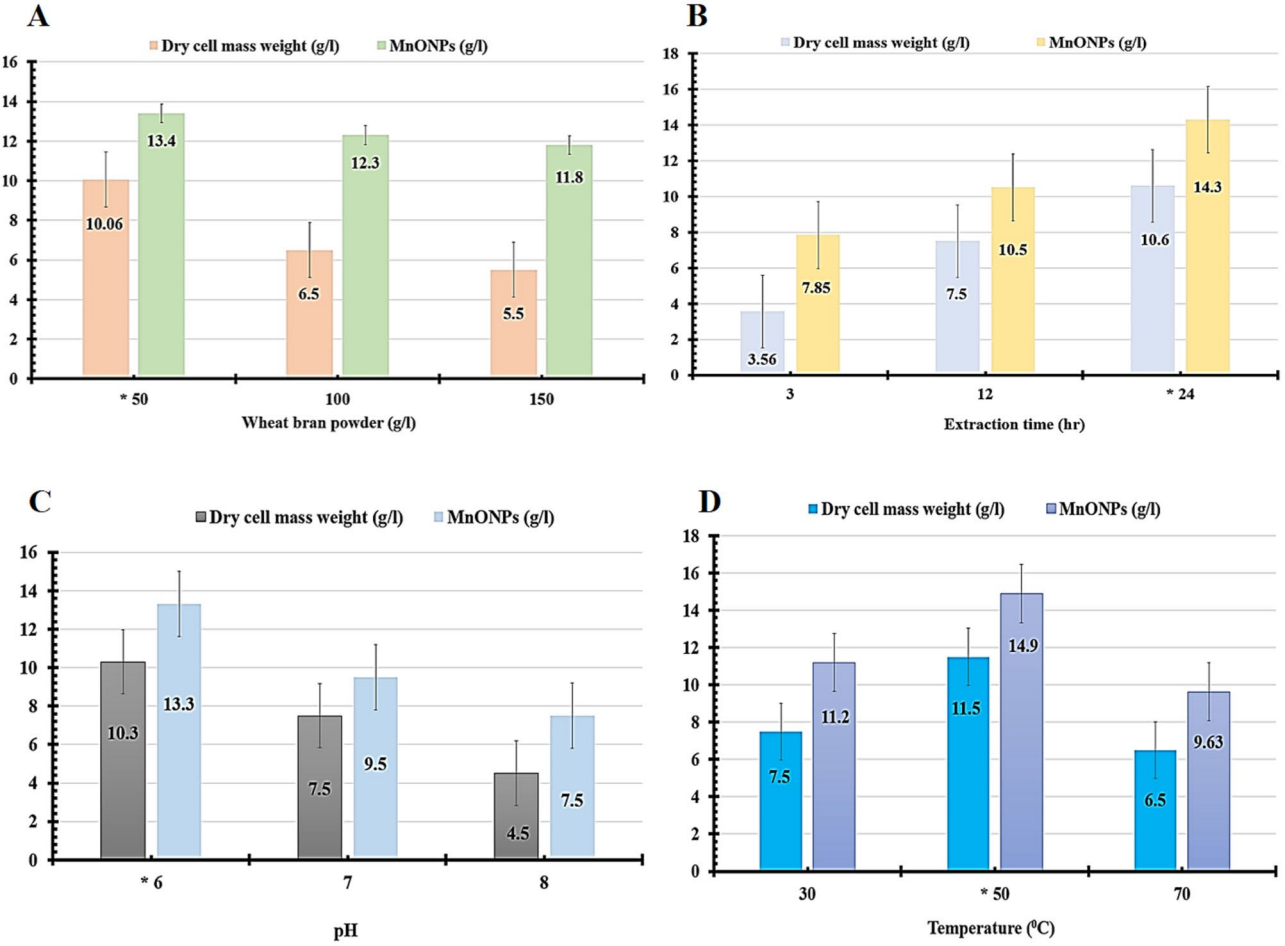
Effective conditions of extraction of soluble nutrients from wheat bran powder that affected the endophytic *Trichoderma* *virens* strain EG92 biomass production, and then fabrication of MnO NPs.

Until now, several approaches have been applied for optimization strategies, such as one variable at a time (OVAT) and statistical designs of experiments (DOE)^19,25,37^. The OVAT strategy is considered a non-efficient and time-consuming method because all variables were studied separately with ignoring interactions among these variables. However, DOE approaches could overcome these issues and reduce the number of trials and the experimental error. Most of the researchers were widely used Plackett–Burman, then Box–Behnken designs for optimizing tested variables. In our attempts, the OVAT method was used to reduce the operating range for all testes variables, then applied DOE. As shown in Fig. 6, the factors that affected the quantities of extracted soluble nutrients using wheat bran powder were studied to maximize the biomass of endophytic strain EG92 and MnO NPs yields. Since the optimized wheat bran weight was 50 g/l, that affected the produced fungal cell mass weight (10.06 g/l) and MnO NPs (13.4 g/l), as shown in Fig. 6A.

Additionally, the maximum fungal cell mass weight (10.5 g/l) and MnO NPs (14.3 g/l) were recorded after 24 h of the extraction process (Fig. 6B). At the same time, the pH of the extraction solution was adjusted at a different value, such as 6, 7, and 8 (Fig. 6C), to distinguish the suitable pH for maximizing the extracted soluble nutrients from wheat bran powder used for fungal cultivation. In this case, the maximum fungal cell mass weight (10.3 g/l) and MnO NPs (13.3 g/l) were detected at pH 6. Also, the optimal temperature used for WB extraction was determined at 50 °C, which produced the maximum fungal cell mass weight (11.5 g/l) and MnO NPs (14.9 g/l) as shown in Fig. 6D. After that, the sufficient conditions for extracting soluble nutrients from wheat bran were prepared finally by mixing 50 g of wheat bran with 1 l of distilled water and pH adjusted at 6 then heated at 50 °C for 24 h. In this condition, the maximum fungal cell mass weight and MnO NPs were recorded at approximately 15.3 g/l and 22.35 g/l, respectively; that increased two-folds than the basal conditions (BC).

Optimization of fermentation settings is a critical restriction for improving bio-refineries economics. Response surface methodology (RSM) is a statistical approach applied to optimize and model several variables. It was used to determine the optimum process conditions by integrating the experimental designs with interpolation using first or second polynomial equations^38–40^. In this work, Plackett–Burman's design was first employed to improve the culturing conditions of the endophytic strain EG92 by statistically detecting the significant variables. Table 3 summarizes the variations in dry cell mass weight from 20.3 to 41.30 g/l, reflecting the importance of investigating the culturing conditions for optimizing the highest biomass productivity. The model coefficient of determination (*R*^2^) points out that 97.73% of the response variability could be described by this model, while 2.27% of the total variation cannot be described.

**Table 3:** Matrix of *Plackett–Burman* experimental design for optimizing the biomass production of endophytic *Trichoderma* *virens* strain EG92, showed the coded and actual values for tested variables besides the experimental and predicted results for dry cell mass weight.

Exp.#	F1	F2	F3	F4	F5	F6	F7	F8	F9	Dry cell mass weight (g/l)	Predicted dry cell mass weight (g/l)
1	+	+	−	+	+	+	−	−	−	31.50	32.13
2	+	−	+	+	+	−	−	−	+	24.60	23.53
3	−	+	+	+	−	−	−	+	−	20.30	21.11
4	+	+	+	−	−	−	+	−	+	21.50	22.13
5	+	+	−	−	−	+	−	+	+	41.30	40.23
6	+	−	−	−	+	−	+	+	−	33.32	34.13
7	−	−	−	+	−	+	+	−	+	31.25	32.06
8	−	−	+	−	+	+	−	+	+	35.09	35.72
9	−	+	−	+	+	−	+	+	+	29.36	28.11
10	+	−	+	+	−	+	+	+	−	36.40	35.15
11	−	+	+	−	+	+	+	−	−	27.30	26.23
12	−	−	−	−	−	−	−	−	−	25.30	24.05

	Inoculums age (h)	Agitation (RPM)	pH	CuSO_4_ (g/l)	Incubation temperature (°C)	Wheat bran extract (%)	Glucose (g/l)	Inoculum’s size (%)	MgSO_4_ (g/l)		
(−)	24	150	6	0.01	25	20	5	1	0.05		
(+)	48	200	7	0.1	30	30	10	4	0.1		

**Figure 7:**
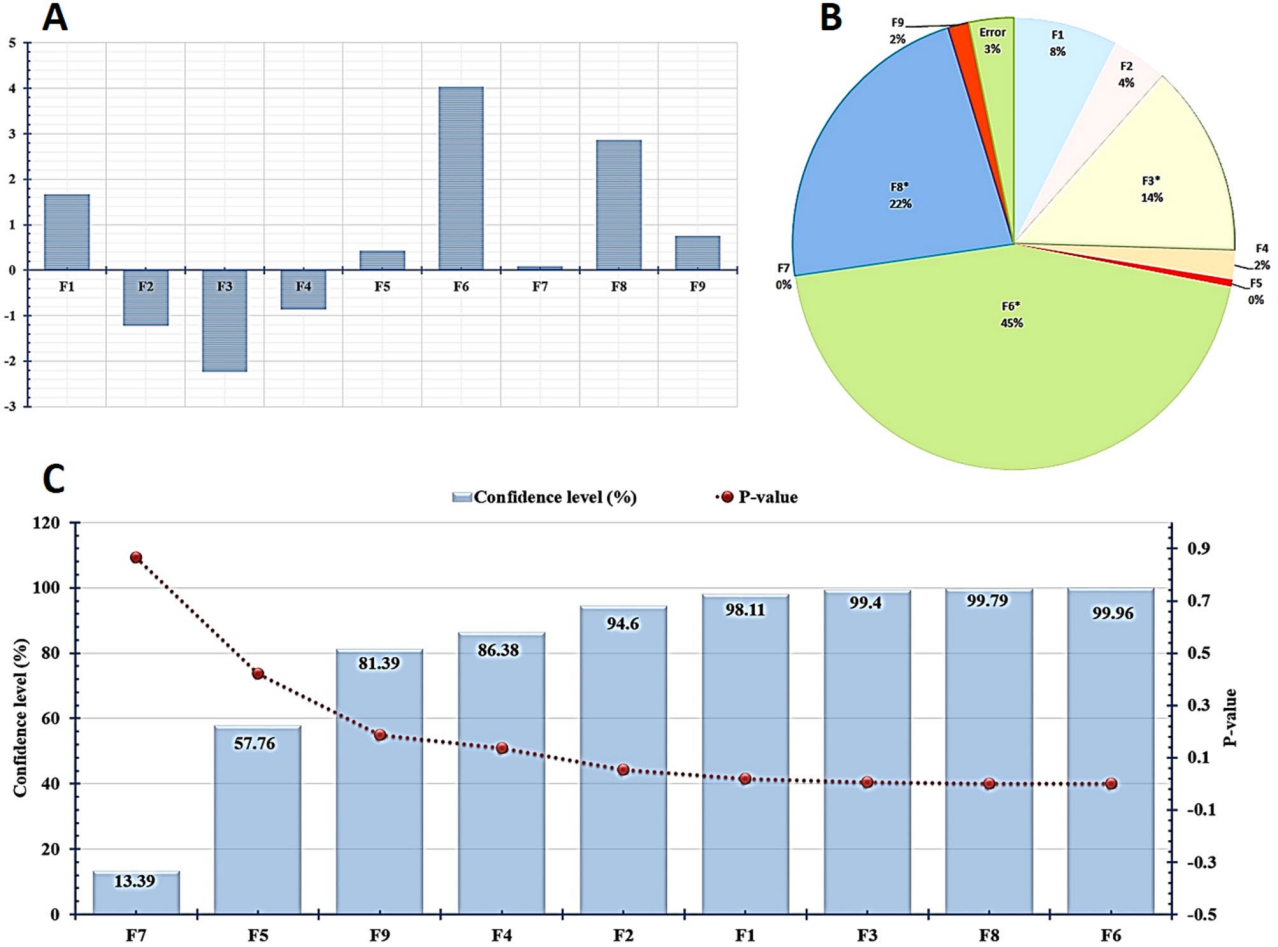
Statistical analysis showing effects of tested variables on dry cell mass of entophytic *Trichoderma* *virens* strain EG92 according to Plackett–Burman design. (**A**) Column chart of calculated main effect of tested variables, (**B**) Pie chart of percent distribution of each variable, and (**C**) Pareto chart represents the calculated *p*-value and confidence levels.

Additionally, the calculated F-ratio was higher than the theoretical one for the regression model, indicating its significance (Table 4). The calculated main effects were graphed as a chart as shown in Fig. 7A. Since all variables positively affect dry cell mass weight except agitation, pH, and CuSO_4_, they have negative effects. Also, different significant variables have the highest presence contribution in this pie chart, such as F3 (14%), F8 (22%), and F6 (45%); thus, these factors are considered as the important variables for increasing the final dry cell mass weight production for endophytic strain EG92 (Fig. 7B).

**Table 4:** Regression statistics and analysis of variance (ANOVA) for *Plackett–Burman* design.

Intercept	Coefficients	Standard error	t Stat	P-value	Confidence level (%)
	29.55	0.44	67.63	1.33863E−08	
F1	1.67	0.49	3.42	0.018934967	98.11
F2	− 1.23	0.49	− 2.51	0.053978572	94.60
*F3	− 2.24	0.49	− 4.58	0.005954457	99.40
F4	− 0.87	0.49	− 1.77	0.136214818	86.38
F5	0.43	0.49	0.87	0.422384502	57.76
*F6	4.04	0.49	8.27	0.000422473	99.96
F7	0.09	0.49	0.18	0.866144618	13.39
*F8	2.86	0.49	5.85	0.002059551	99.79
F9	0.75	0.49	1.53	0.186109542	81.39

ANOVA					
	df	SS	MS	F	Significance F
Regression	9	423.30	47.03	16.43	0.00332
Residual	5	14.32	2.86		

Regression statistics					
	Multiple R	R Square	Adjusted R Square	Standard Error	Observations
	0.98	0.97	0.91	1.69	15

*Denotes a statistically significant difference between the test factors.

Furthermore, the graphed *Pareto* chart shows the ranking of each estimated variable using confidence level (%) and *p*-value (Fig. 7C). Non-significant and significant variables were detected simply by the calculated confidence level (%) since the significant variables have confidence levels up to 95%, such as inoculum age (98.11%), pH (99.4%), inoculum size (99.96%), and WBE (99.79%). Finally, this improvement was achieved at inoculums age (48 h), agitation (150 RPM), pH 6, CuSO_4_ (0.01 g/l), incubation temperature (30 °C), WBE (30%), glucose (10 g/l), inoculums size (4%), and MgSO_4_ (0.1 g/l). These conditions increased the maximum dry cell mass of the endophytic strain EG92 up to six-fold (42.68 g/l) than using the BC. Subsequently, the fungal bioactive metabolites were used to fabricate MnO NPs that increase up to five-folds (55.29 g/l).

Secondly, the selected three independent factors (pH, WBE, and inoculums size) were optimized statistically via Box–Behnken design to produce maximum fungal biomass weight. Since the levels of the most significant variables were varied from low (−), middle (0), and high (+) values, whereas the non-significant variables stabilized at their values. Table 5 represents the design matrix for tested variables indicating both coded and actual values besides experimental and predicted dry cell mass weights. The optimization of significant variables affecting the dry cell mass weights was achieved via the regression equation that generated relating the response variable to coded levels of independent variables (Table *8). These results show that three independent variables significantly increase the quantities of dry cell mass. Since, there is a variation in dry cell mass weight from 58.18 g/l (trail 2) to 86.89 g/l (trail 13). This model is significant at a high confidence level because the F-value was several times higher than the tabulated one. The probability *p*-value was also very low (*p* ≤ 0.05). The model coefficient of determination (*R*^2^) points out 96.47% of the response variability, which this model could designate. At the same time, 3.53% of total variation cannot be defined (Table 6). High correlation (Adj. *R*^2^ = 0.90) also indicated the degree of accuracy that confirmed the high significance of the model.

**Table 5:** Matrix of *Box–Behnken* experimental design for optimizing the biomass production of entophytic *Trichoderma* *virens* strain EG92, showing the experimental and theoretical values of dry cell mass weight under tested variables.

# Trial	X1	X2	X3	Dry cell mass weight (g/l)	Predicted dry cell mass weight (g/l)
1	0	0	0	71.20	70.77
2	−	−	0	58.18	61.27
3	0	+	+	70.27	71.84
4	0	−	+	61.74	60.85
5	0	0	0	70.05	70.77
6	+	+	0	62.68	59.58
7	+	0	+	81.19	82.71
8	+	0	−	83.63	85.85
9	−	+	0	66.56	67.21
10	−	0	+	83.97	81.76
11	0	+	−	60.93	61.81
12	0	−	−	79.18	77.61
13	−	0	−	86.89	85.36
14	0	0	0	71.05	70.77
15	+	−	0	70.99	70.34

	pH	WBE (%)	Inoculum’s size (%)		
−	4	30	4		
0	5	40	8		
+	6	50	12

**Table 6:** Regression statistics and analysis of variance (ANOVA) for *Box–Behnken* experimental design.

Intercept	Coefficients	Standard error	t Stat	P-value	Confidence level (%)
	70.77	1.67	42.46	1.36792E−07	
X1	0.36	1.02	0.35	0.738184524	26.18
X2	− 1.20	1.02	− 1.18	0.291086336	70.89
X3	− 1.68	1.02	− 1.65	0.15993542	84.01
*X1X2	− 4.17	1.44	− 2.89	0.034116006	96.59
X1X3	0.12	1.44	0.08	0.938873008	6.11
*X2X3	6.70	1.44	4.64	0.005640579	99.44
*(X1)2	4.86	1.50	3.24	0.023068068	97.69
*(X2)2	− 11.03	1.50	− 7.34	0.000736351	99.93
*(X3)2	8.29	1.50	5.52	0.002676051	99.73

ANOVA					
	df	SS	MS	F	Significance F
Regression	9	1139.186386	126.5762651	15.18585511	0.003986807
Residual	5	41.6757121	8.335142421		
Total	14	1180.862098			

Regression statistics					
	Multiple R	R square	Adjusted R square	Standard error	Observations
	0.98	0.96	0.90	2.89	15

*Denotes a confidence level that is statistically significant (the significant variables have confidence levels up to 95%).

**Figure 8:**
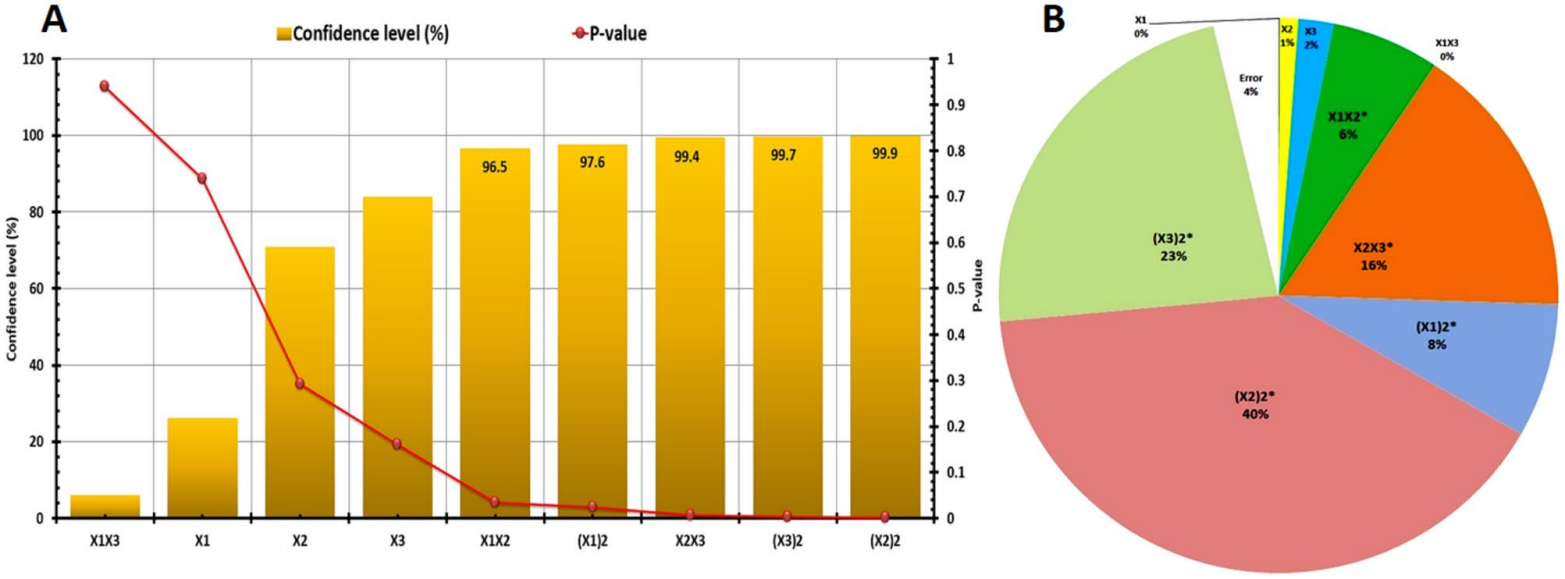
Statistical analysis of effects of tested significant variables on dry cell mass of entophytic *Trichodermavirens* strain EG92, according to Box–Behnken experimental design. (**A**) Pareto chart represents the calculated *p*-value and confidence level (%), and (**B**) Pie chart shows percent distribution of each variable.

Additionally, the *Pareto* chart (Fig. 8A) was graphed using confidence level (%), and *p*-values showed the ranks of each variable; since the significant variables have confidence levels up to 95%. ANOVA determined the percent contribution of each variable and the interactions among tested variables. Figure 8B shows the highest presence contribution of different variables, which were recorded at X_2_^2^ (40%), followed by X_3_^2^ (23%), and X_1_^2^ (8%). Additionally, the highest presence contributed to interaction effects of significant variables was recorded among X_2_X_3_ (16%), and X_1_X_2_ (6%). So, these variables and their mixture are important for improving the produced dry cell mass weight for endophytic strain EG92. A second-order polynomial model was fitted by Eq. (4), and the actual values were calculated using Eq. (5) to predict the optimal point*.* In conclusion, this improvement was achieved at pH 5.5, WBE (35%), and inoculums size (10%), which increased the maximum dry cell mass of endophytic strain EG92 up to 12-fold (89.63 g/l) than using BC. Subsequently, the fungal bioactive metabolites were used to fabricate MnO NPs that increased up to 8-folds (82.93 g/l).

### Large-scale fermentation strategies for scaling-up production of endophytic strain EG92 and MnO NPs yields

**Figure 9:**
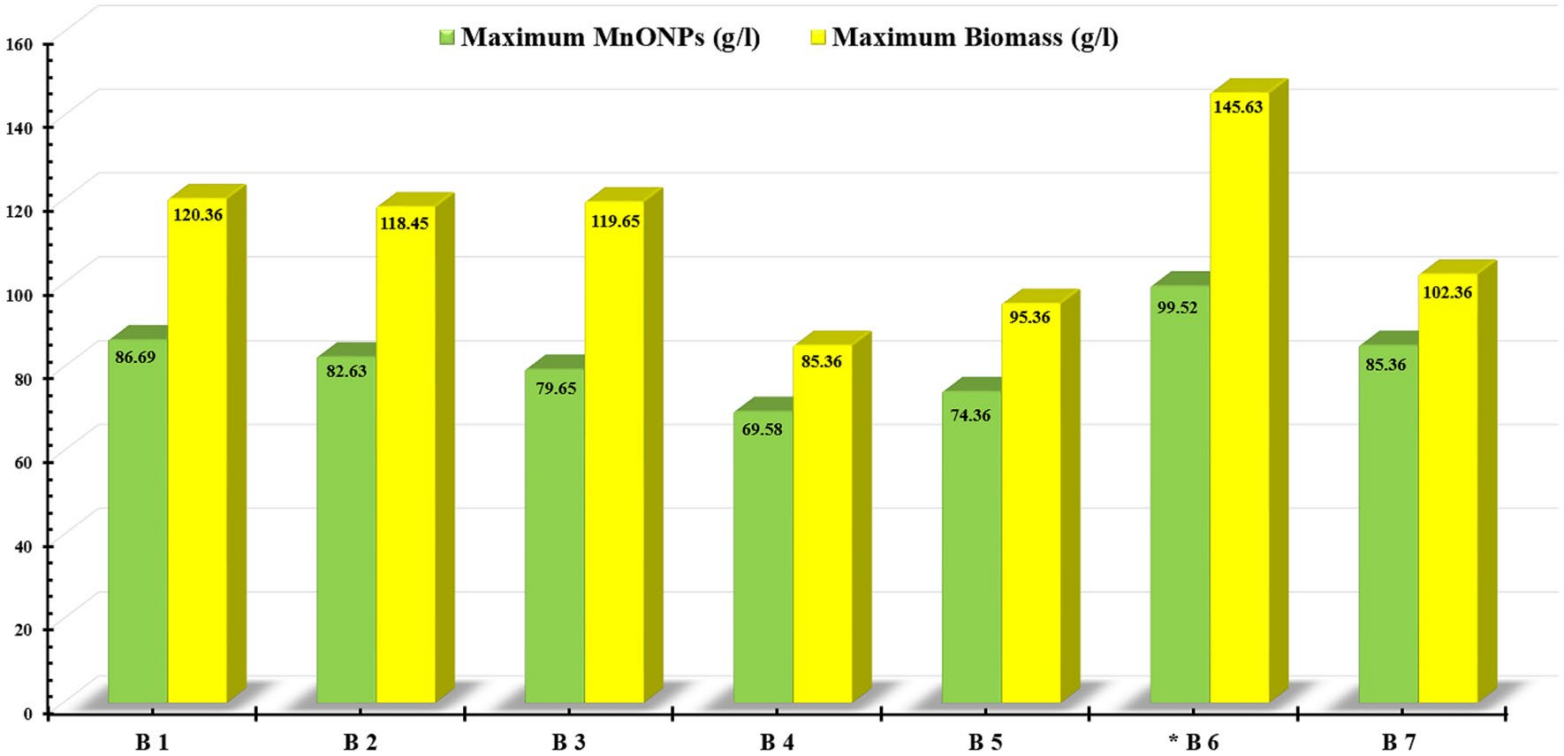
Effects of different aeration and agitation regimes and its timing on the growth of endophytic *T.virens* strain EG92 hence the fabricated MnO NPs mass weight using submerged fermentation system via batch mode within 7 L bioreactor. (B1); airflow started from 1 to 4.5 vvm/12 h and agitation started from100 to 800 rpm/6 h, (B2) airflow started from 0.5 to 2 vvm/6 h and agitation started from 100 to 800 rpm/3 h, (B3) airflow started from 1.5 to 3.5 vvm/12 h and agitation started from 50 to 250 rpm/6 h, (B4) airflow started from 0.5 to 2.5 vvm/6 h and agitation constant at 400 rpm, (B5) airflow constant at 2 vvm/12 h and agitation started from 50 to 300 rpm/6 h, (B6) airflow and agitation adjusted to let the dissolved O_2_ level did not below 10%, and (B7) airflow and agitation adjusted to let the dissolved O_2_ level did not below 40%.

**Figure 10:**
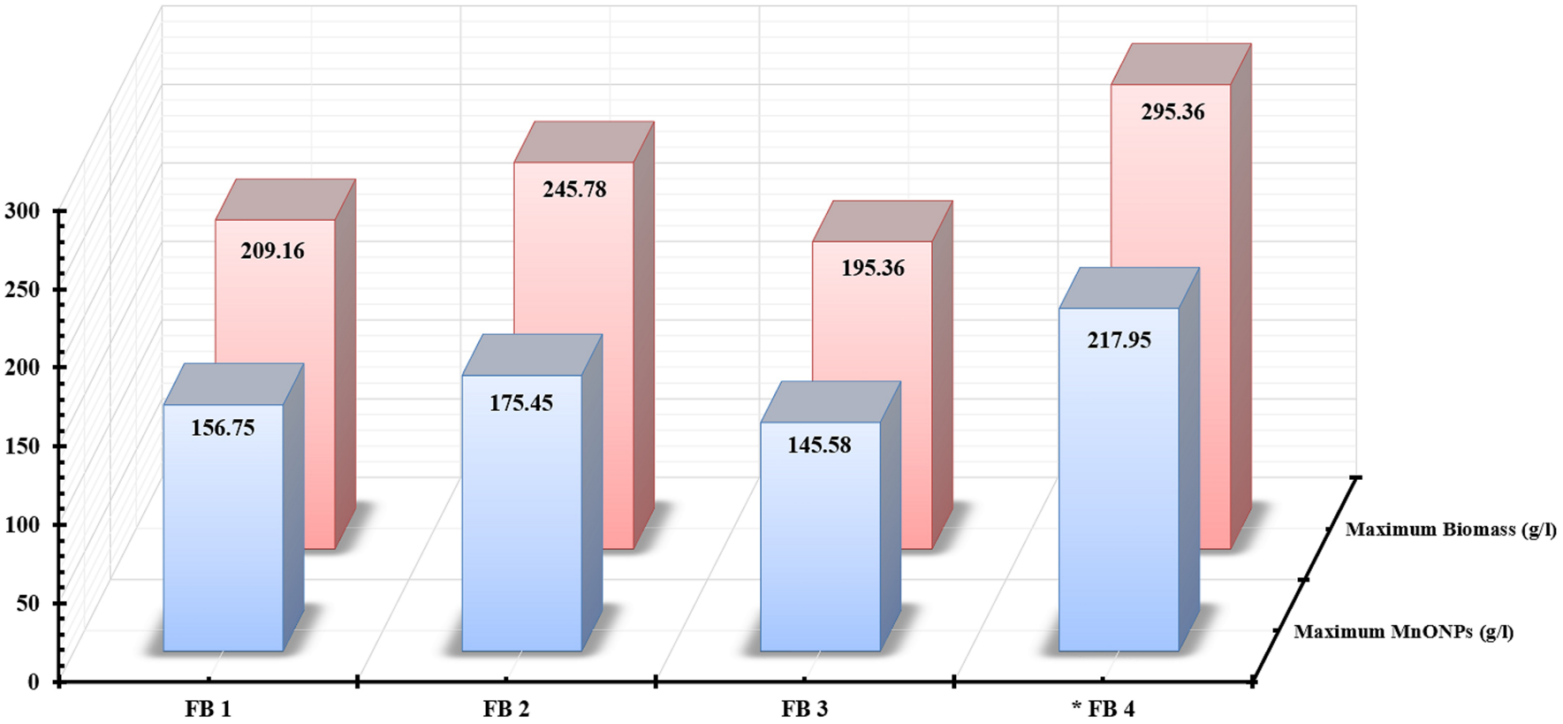
Study the effects of different fed-batch fermentation modes on the growth of endophytic *T.* *virens* strain EG92 hence the fabricated MnO NPs mass weight using submerged fermentation system via fed-batch mode within 7 L bioreactor. (FB1) constant feeding for whole medium, (FB2) Exponential pulses feeding for whole medium, (FB3) constant feeding for WBE, and (FB4) exponential pulses feeding for WBE.

In this work, the submerged fermentation system was used to study the growth kinetics for endophytic strain EG92 via batch fermentation mode within 7 L bioreactor. Since the submerged fermentation system was considered a simplistic process that provided improved control. Meanwhile, the scaling-up production of filamentous fungi was influenced by many parameters like agitation speed and airflow rates. So, to maximize the endophytic strain, EG92 dry cell mass weight hence gets the best MnO NPs yield. The mixing characteristics of different aeration/agitation rates and timing were studied via seven control strategies in batch fermentation (BF) modes (Fig. 9). The kinetics results showed variations in *X*_*max*_ and *P*_*max*_*,* which indicated the highest physiological activities depending on different aeration/agitation rates and timing. As shown in Fig. 9, the used aeration/agitation regime in the B6 case (airflow and agitation adjusted to let DO level did not below 40%) produced the highest *X*_*max*_ (145.63 g/l) and *P*_*max*_ (99.52 g/l) after 162 h by the side of μ_max_ and Y_X/S_ were recorded as 0.084, and 7.65, respectively. Therefore, the adjustment of DO level at ≥ 10% is considered an important factor affecting the growth of the endophytic strain EG92, which increases up to 21.08% (145.63 g/l) than the BC. Subsequently, the fabricated MnO NPs using these concentrations of bioactive metabolites increased up to ten-folds (99.52 g/l) than the basal reducing/capping agent’s concentration. At the endpoint of the batch fermentation mode, the tested four feeding strategies were started by different fed-batch fermentation modes. These tested four fed-batch fermentation modes (FBF) were set up by the optimized medium ingredients or WBE, only to scaling-up the production of fungal growth. As shown in Fig. 10, different feeding strategies were utilized to explain the behavior of endophytic strain EG92 cells and the produced MnO NPs. The highest data were recorded by feeding WBE via exponential pulses feeding regime (FB4). Since, *X*_*max*_, and *P*_*max*_ were recorded as 295.36 and 217.95 g/l, respectively after 48 h at μ_max_ (0.082) and Y_X/S_ (20.69). These results are regarded as unique in that they maximize endophytic *Trichoderma* biomass production and thereby MnO NP biosynthesis using waste material, which has never been done before to our knowledge.

### Optimization of myco-synthesized MnO NPs reaction

An optimization technique is employed during the biosynthesis of any nanomaterials to find the ideal experimental conditions for producing the best and most stable results. Traditional optimization systems ignore interactions between independent variables, and running many experiments is time-consuming and expensive. In our work, the effect of different parameters on biomass production and MnO NPs yield could be measured by statistical experimental design (*Taguchi* method) to find the best conditions using orthogonal arrays and analysis of variance. Taguchi's experimental design, also known as a robust parameter design, can compare different experimental conditions and select the one with the least variability as the strongest.

**Figure 11:**
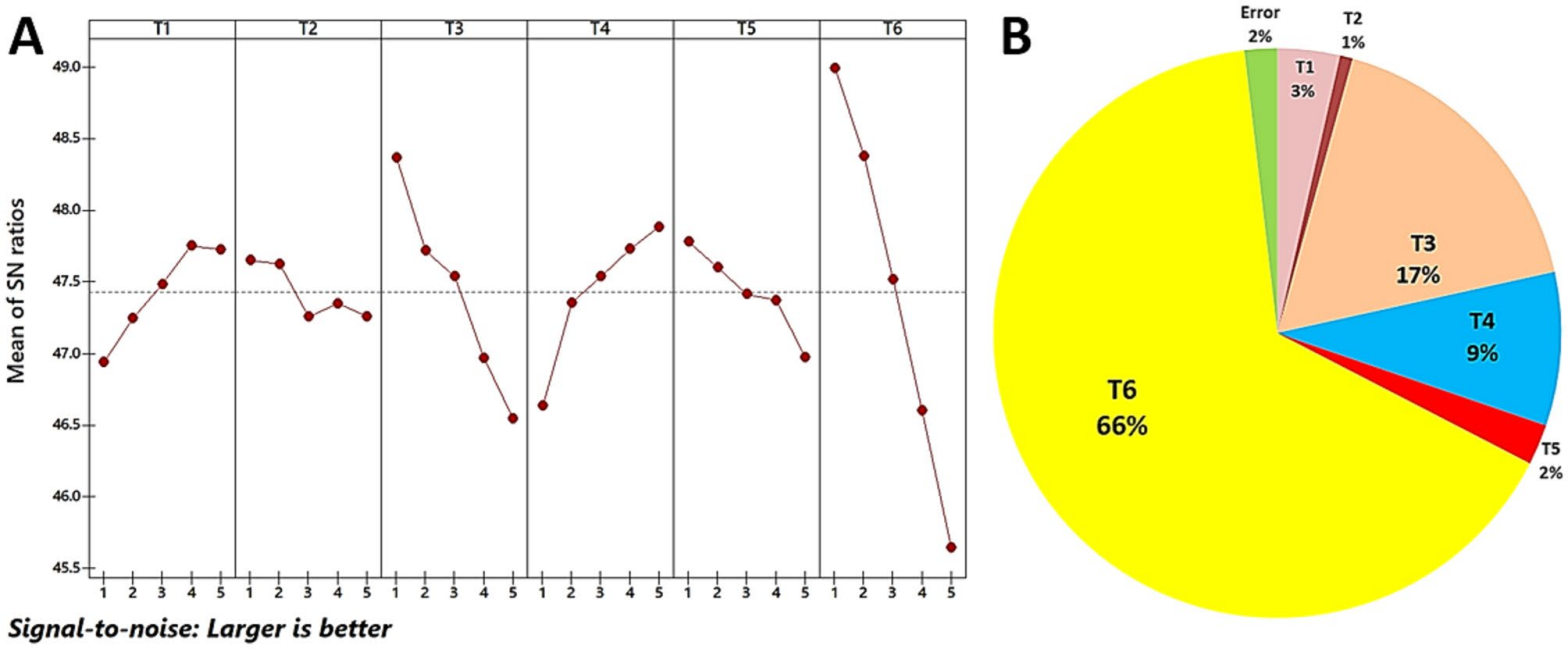
A statistical evaluation of the influence of indicated significant factors including metal Precursor conc. (V1), reducing/Capping agent conc. (V2), temperature (V3), pH (V4), stabilizing agent conc. (V5), and reaction time (V6) on the biogenic production of MnO NPs was carried out using the Taguchi experimental design. (**A**) Response graph of S/N ratio for fungus-fabricated MnO NPs, (**B**) Pie chart shown percent distribution of each factor.

So, the Taguchi design method is a statistical optimization tool that provides simple, efficient, and systematic results to maximize production^26,41^. For studying the most influencing variables that optimized the production of MnO NPs using fungal extract as reducing/capping agents and MnCl_2_·4H_2_O as a parent compound (precursor), a Taguchi experimental design (TD) was planned with 25 trails using 6 factors at five levels. Then the fungus-fabricated MnO NPs mass weight (model response) was determined to calculate the largest S/N ratios for each trial (Fig. 11G). Generally, there are variations in MnO NPs mass weights increases from 160.45 ± 1.99 g/l (Trail 5) to 315.3 ± 0.19 g/l (Trail 19), so the tested variables increase the quantities of MnO NPs weights significantly as listed in (Table 7).

**Table 7:** Matrix of Taguchi experimental design for optimizing the MnO NPs biogenic production using endophytic *Trichoderma* *virens* strain EG92 extract.

Trail	V1	V2	V3	V4	V5	V6	MnONPs (g/l)	Predicted MnONPs (g/l)	S/N ratio (dB)
T1	1	1	1	1	1	1	289.36 ± 4.21	293.58	49.23
T2	1	2	2	2	2	2	265.36 ± 4.7	260.79	48.48
T3	1	3	3	3	3	3	225.36 ± 2.7	228.01	47.06
T4	1	4	4	4	4	4	195.36 ± 0.13	195.23	45.82
T5	1	5	5	5	5	5	160.45 ± 1.99	162.45	44.11
T6	2	1	2	3	4	5	200.09 ± 0.04	200.05	46.02
T7	2	2	3	4	5	1	280.69 ± 0.18	280.87	48.96
T8	2	3	4	5	1	2	262.36 ± 7.1	269.46	48.38
T9	2	4	5	1	2	3	193.89 ± 1.41	195.29	45.75
T10	2	5	1	2	3	4	226.36 ± 5.55	220.81	47.1
T11	3	1	3	5	2	4	240.24 ± 1.3	241.5	47.61
T12	3	2	4	1	3	5	170.28 ± 2.94	167.34	44.62
T13	3	3	5	2	4	1	247.34 ± 0.89	248.15	47.87
T14	3	4	1	3	5	2	280.25 ± 6.58	273.66	48.95
T15	3	5	2	4	1	3	261.36 ± 0.89	262.25	48.34
T16	4	1	4	2	5	3	225.9 ± 5.7	220.19	47.08
T17	4	2	5	3	1	4	216.36 ± 7.6	208.78	46.7
T18	4	3	1	4	2	5	229.23 ± 5.06	234.29	47.21
T19	4	4	2	5	3	1	315.3 ± 0.19	315.11	49.97
T20	4	5	3	1	4	2	245.36 ± 4.41	240.94	47.8
T21	5	1	5	4	3	2	260.3 ± 1.34	261.64	48.31
T22	5	2	1	5	4	3	293.45 ± 6.3	287.15	49.35
T23	5	3	2	1	5	4	194.3 ± 18.68	212.98	45.77
T24	5	4	3	2	1	5	205.36 ± 3.8	201.58	46.25
T25	5	5	4	3	2	1	280.203 ± 2.2	282.39	48.95

	Metal precursor conc. (M)	Reducing/capping agent conc. (%)	Temperature (°C)	pH	Stabilizing agent conc. (%)	Reaction time (h)			
L1	1.25	100	60	9	20	5			
L2	1	80	50	8	15	4			
L3	0.75	60	40	7	10	3			
L4	0.5	40	30	6	5	2			
L5	0.25	20	20	5	0	1			

The regression equation was generated by the response (dependent variable) and the coded levels of independent variables (Table 8). The model coefficient of determination R^2^ = 0.9814, which points out that this model's 98.14% of the response variability could be designated, while 1.68% of the total variation cannot be defined. High correlation (Adj. *R*^2^ = 0.9751) also indicates the degree of accuracy that confirmed the high significance of the model. Since the model was significant because F-value was higher than the tabulated several times. Additionally, the calculated confidence level (%) and *p*-values showed the ranks of each variable since the significant variables have confidence levels up to 95%. Figure 11H shows the highest presence contribution for different variables were recorded at reaction time (66%), followed by reaction temperature (17%) and reaction pH (9%). So, these variables and their mixture are considered important for increasing MnO NPs mass weight using endophytic *T.* *virens* strain EG92 extract. Finally, the improvement of the fungus-fabricated MnO NPs reaction was achieved at MnCl_2_·4H_2_O as a parent compound (0.25 M), fungal extract as reducing/capping agents (100%), reaction pH ~ 5, reaction temperature (60 °C), reaction time (5 h), and fungal extract as stabilizing agent (20%). So, the maximum fungus-fabricated MnO NPs mass weight was increased up to 40-fold (395.36 g/l) than the BC. Finally, this study is considered the first study that fabricated MnO NPs industrially using extracted bioactive metabolites using endophytic *Trichoderma* *virens* through statistical bioprocessing strategies. Also, the fabricated MnO NPs were prepared biologically using the extracellular bioactive metabolites of endophytic strain EG92 as a capping/reducing agent and MnCl_2_·4H_2_O as a parent compound.

**Table 8:** Regression statistics and analysis of variance (ANOVA) for Taguchi experimental design.

Intercept	Coefficients	Standard Error	t Stat	P-value	Confidence level (%)
	321.07	6.75	47.54	2.23113E−20	
V1	5.28	0.90	5.86	1.51738E−05	99.98
V2	− 2.41	0.90	− 2.67	0.015746934	98.42
V3	− 11.66	0.90	− 12.92	1.52472E−10	99.98
V4	8.28	0.90	9.17	3.32135E−08	99.98
V5	− 4.27	0.90	− 4.74	0.000164547	99.98
V6	− 22.72	0.90	− 25.18	1.75391E−15	100

ANOVA					
	df	SS	MS	F	Significance F
Regression	6	38,629.40865	6438.234775	158.1133916	1.43084E−14
Residual	18	732.9437737	40.71909854		
Total	24	39,362.35242			

Regression statistics					
	Multiple R	R square	Adjusted R square	Standard error	Observations
	0.99	0.98	0.98	6.38	25

**Figure 12:**
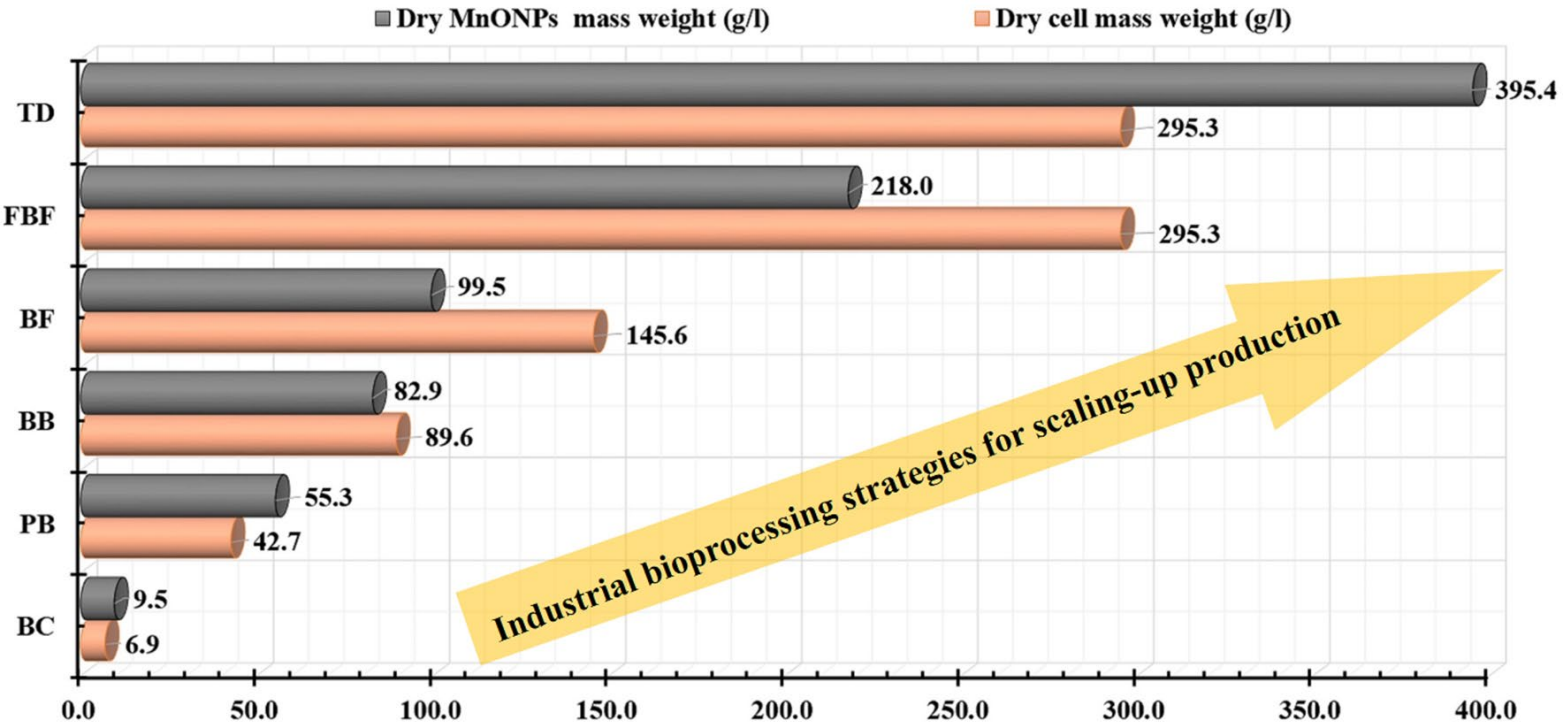
Summary for the bioprocessing strategies that applied for optimizing the endophytic *Trichoderma* *virens* strain EG92 mass weight (g/l), as well as the fungus-fabricated MnO NPs mass weight (g/l) using Plackett–Burman (PB), Box–Behnken (BB), and Taguchi (TD) experimental designs. Scaling-up production of fungal mass production via semi-industrial bioreactor (7 L) by submerged fermentation system through batch (BF) fed-batch (FBF) fermentation modes that compared finally to the basal conditions (BC).

Figure 12 displays the statistical bioprocessing strategies applied for scaling up production of the endophytic strain EG92 mass weight and the fungus-fabricated MnO NPs mass weight. These experiments were designed to optimize the culturing conditions for the endophytic strain EG92 using PB, and BB designs. These cells, and hence MnO NPs, were then scaled up in a semi-industrial bioreactor utilizing a submerged fermentation system in BF and FBF modes. Since the BF cultivation system was enhanced using multiple aeration/agitation rates, FBF mode was also improved, using various feeding strategies for the whole medium or WBE that used exponential or pulses mode. Additionally, the MnO NPs fabrication reaction was also optimized by TD. Generally, these statistical strategies have an important advantage over conventional methods. The final results indicated that dry cell mass and fungus-fabricated MnO NPs productions increased, as shown in Fig. 12.

To date, no study has looked into the biosynthesis of MnO NPs using endophytic *Trichoderma* *virens* extract as a reducing, capping, and stabilizing agent, followed by industrial optimization of operational variables using various statistical experimental methodologies and then scaling-up production via fed-batch fermentation.

### Application of myco-synthesized MnO NPs as an anti-phytopathogenic agent in vitro

Different concentrations of fabricated MnO NPs were prepared to evaluate its antagonistic activities and detect MIC, MBC, and MFC using different phytopathogens. Table 9 displays the diameter of inhibition zones that express as millimeters. Overall, the excellent MIC for myco-synthesized MnO NPs was recorded against phytopathogenic bacteria at 210 µg/ml, however against phytopathogenic fungi was verified at 330 µg/ml. In the case of phytopathogenic fungi, the largest inhibition zones were recorded against *Alternaria* *alternate* (42.75 ± 3.96), followed by *Helminthosporium* sp., (40.38 ± 2.58), however, the lowest one measured against *Phytophthora* *arenaria* at (28.13 ± 2.97). However, against phytopathogenic bacteria, the largest inhibition zones were recorded against *Erwinia* *amylovora* (50.62 ± 2.34), followed by *Acetobacter* *pasteurianus* (47.81 ± 6.34) and *Erwinia* *carotovora* (42.18 ± 2.56). The lowest results were noted against *Clavibacter* *michiganensis* (33.75 ± 1.90) *Erwinia* *persicina* (39.37 ± 1.02). Also, the obtained values of MBC were ranged between 200 and 280 µg/ml, and the MFC values extended to 340–440 µg/ml. Generally, the myco-synthesized MnO NPs have rapid and more effective antagonistic activities against phytopathogenic bacteria than fungi in this study. Because our myco-synthesized MnO NPs have quicker and more accurate antagonistic activities against phytopathogenic bacteria than fungi, they may be used as an alternative nano-bio-pesticide in the future to control various disease-pathogens. MnO NPs have several unique properties, such as the ability to quickly penetrate pathogen cells by inducing cell membrane distortions and destruction, resulting in microbial cell death. Joshi et al. biosynthesized MnO_2_ NPs using plant extract (leaves extract of *Datura* *stramonium* as reducing agent), then 10 mg/ml of MnONPs was checked against human pathogens and discovered that they exhibited effective antagonistic effects (30-44 mm)^24^. To biosynthesize ZnO, MnO_2_, and MgO NPs, Ogunyemi et al*.*, employed rhizospheric bacteria (*Paenibacillus* *polymyxa* strain Sx3). Their studies revealed significant inhibitory effects (13–17 mm) at a 16.0 µg/ml concentration against phytopathogenic bacteria (*Xanthomonas* *oryzaepv.oryzae*)^6^. The application to rice plants also enhances plant growth factors and biomass while lowering the expression of rice bacterial leaf blight disease, according to this study. So, these metallic nanoparticles are non-toxic, bio-safe, and bio-compatible at low doses. Finally, the field application of mycosynthesized MnO NPs as a potential pesticide in agriculture has yet to be thoroughly investigated until now.

**Table 9:** Bio-application of the fabricated MnO NPs using the endophytic *Trichodermavirens* strain EG92against phytopathogens in vitro to detect MIC, MFC, and MBC values.

Pathogens	Fungus-fabricated MnO NPs concentration (µg/ml)											
	Zone of inhibition (mm)											
	10	50	90	130	170	210	250	290	330	370	MFC (µg/ml)	MBC (µg/ml)
Phytopathogenic fungi												
*Fusariumsolani*	9.65 ± 1.34	11.51 ± 3.98	15.38 ± 4.89	16.88 ± 2.89	22.50 ± 2.90	13.5 ± 1.1	18.90 ± 2.89	28.75 ± 2.89	32.5 ± 1.8	21.90 ± 2.90	400	
*Fusariummoniliforme*	16.59 ± 2.09	13.54 ± 2.78	13.59 ± 1.29	21.09 ± 1.78	28.13 ± 2.67	16.44 ± 2.82	25.62 ± 2.78	33.19 ± 1.76	37.88 ± 3.25	33.25 ± 3.56	340	
*Helminthosporium* sp.	19.95 ± 1.98	18.56 ± 2.54	16.41 ± 3.67	23.91 ± 1.89	31.88 ± 1.04	20.06 ± 2.4	20.37 ± 1.89	35.06 ± 1.34	40.38 ± 2.58	38.5 ± 2.89	420	
*Alternaria* *alternate*	19.52 ± 2.67	13.25 ± 1.67	17.81 ± 3.65	25.31 ± 0.97	33.75 ± 2.09	21.13 ± 3.05	21.25 ± 1.45	37.13 ± 3.98	42.75 ± 3.96	38.5 ± 1.89	360	
*Aspergillus* *flavus*	13.65 ± 0.45	16.95 ± 1.89	19.69 ± 5.89	19.69 ± 2.89	26.25 ± 4.23	16.88 ± 1.51	23.75 ± 1.89	31.88 ± 2.98	36.25 ± 1.63	28 ± 2.98	380	
*Aspergillus* *niger*	11.85 ± 2.87	16.65 ± 4.98	19.41 ± 1.78	19.41 ± 2.98	25.88 ± 5.89	15.56 ± 2.73	23.37 ± 1.42	30.56 ± 1.25	34.875 ± 4.64	24.5 ± 1.23	400	
*Phytophthoraarenaria*	10.95 ± 1.09	12.75 ± 0.45	14.34 ± 6.90	14.34 ± 2.56	19.13 ± 2.89	12.44 ± 1.78	16.62 ± 1.90	24.93 ± 2.75	28.13 ± 2.97	22.75 ± 3.67	440	
Phytopathogenic bacteria												
*Clavibactermichiganensis*	8.14 ± 0.05	14.30 ± 1.89	16.50 ± 0.46	20.50 ± 2.90	26.91 ± 2.89	33.75 ± 1.90	30.25 ± 2.90	28.63 ± 3.89	24.69 ± 2.12	20.71 ± 2.89		220
*Erwiniacarotovora*	10.73 ± 2.98	18.85 ± 2.67	21.75 ± 0.56	28.13 ± 1.89	38.81 ± 3.90	42.18 ± 2.56	41.5 ± 1.56	35.23 ± 2.12	35.42 ± 1.93	19.69 ± 2.89		200
*Acetobacterpasteurianus*	9.25 ± 2.97	16.25 ± 1.89	18.75 ± 3.56	22.88 ± 3.03	28.69 ± 2.54	47.81 ± 6.34	38.5 ± 3.89	31.59 ± 2.89	30.99 ± 3.12	22.31 ± 2.89		240
*Erwiniaamylovora*	8.51 ± 3.13	14.95 ± 2.89	17.25 ± 1.09	23.75 ± 2.05	34.41 ± 1.78	50.62 ± 2.34	48.75 ± 2.45	49.23 ± 3.45	48.96 ± 1.34	40.28 ± 2.16		280
*Erwiniapersicina*	9.62 ± 2.45	16.90 ± 1.34	19.50 ± 2.89	16.25 ± 1.65	29.34 ± 3.56	39.37 ± 1.02	36.5 ± 1.89	22.63 ± 1.23	21.92 ± 4.89	16.01 ± 5.89		200

## Conclusions

In this study, biologically synthesized rod-shaped myco-synthesized MnO NPs with average crystallite size of ~ 35 nm were formed utilizing MnCl_2_.4H_2_O (precursor), and endophytic *Trichoderma* *virens* strain EG92 extract (reducing, stabilizing, and capping agents) that cultivated in Wheat bran medium. Multiple statistical optimization approaches (Placket–Burman and Box–Behnken designs) were used to achieve the highest fungal cell mass weight. Since the Placket–Burman design was used to screen the major influences on fungal biomass production. The correlations between the important parameters and the produced fungal biomass weight were then correlated using a Box–Behnken factorial design. The yields of EG92 cells and MnO NPs were then industrially boosted using a submerged fermentation batch mode with adjustable aeration/agitation rates, followed by a fed-batch fermentation mode using an exponentially pulsed feeding system. After 48 h, this industrial production line yielded X_max_ (295.36 g/l) and P_max_ (217.95 g/l). The MnO NPs yield was increased 40-fold (395.36 g/l) above baseline conditions using Taguchi design to improve the fabrication reaction. These results are unique in that they maximize endophytic Trichoderma biomass production and, as a result, MnO NPs biosynthesis using waste material, which has never been done before. This level of dry weight biomass and MnO NPs yield productivity has never been seen before. The antagonistic activities against phytopathogens were investigated, and *Erwinia* *amylovora* (50.62 ± 2.34) had the highest inhibition zones, followed by *Alternaria* *alternate* (42.75 ± 3.96). Furthermore, MBC values for bacterial cells varied from 200 to 280 µg/ml, whereas MFC values for phytopathogenic fungus cells ranged from 340 to 440 µg/ml. Finally, these myco-synthesized MnO NPs are non-toxic, bio-safe, and bio-compatible at low doses. As a result, it could be employed as nano-bio pesticides in agriculture.

## Materials and methods

### Materials

Healthy Egyptian Black Eggplant (*Solanum* *melongena* L.) leaves were collected from the experimental farm of City of Scientific Research and Technological Applications (SRTA-City), New Borg El-Arab City, Alexandria, Egypt. The tested phytopathogenic fungi (*Fusarium* *solani,* *Fusarium* *moniliforme,* *Helminthosporium* sp., *Alternaria* *alternate*, *Aspergillus* *flavus*, *Aspergillus* *niger*, and *Phytophthora* *arenaria*), and bacteria (*Clavibacter* *michiganensis*, *Erwinia* *carotovora,* *Acetobacter* *pasteurianus,* *Erwinia* *amylovora,* and *Erwinia* *persicina*) were collected from Department of Bioprocess Development, GEBRI, SRTA-City, Egypt and Zagazig University, Faculty of Agriculture, El-Sharkia, Egypt.

### Collection of plants raw materials and isolation of mycoentophyte

Plant leaves were washed with tap water three times, then immersed successively in 70% ethanol for 5 min, 99% ethanol for 2 min, and the leaves were finally soaked in a 6% sterilization solution composed of (10% NaHCO_3_: 0.1% Tween-20) for 2 min. The sterilized leaves were washed with sterilized distilled water several times and allowed to dry in a laminar flow cabinet^33^. The final rinse water was spread on potato dextrose agar (PDA) plates containing 200 g of potatoes, 20 g of dextrose, and 20 g of agar to test the sterilising step. These sterilized leaves were cut and grinded with a sterile scalpel blade and crushed in a mortar. Using the serial dilution method, 100 µl of the diluted leaves extract was prepared and spread on PDA plates and incubated at 30 °C for three weeks. Entophytic fungi were selected and purified using PDA plates. Finally, the purified entophytic fungi were grown using an MGYP broth medium that composed of 0.3% malt extract, 1% glucose, 0.3% yeast extract, 0.5% peptone, and the final pH was adjusted at 5.9. Then these cultures were incubated at 30 °C under shaking conditions (150 rpm) for 72 h.

### Biosynthesize of MnO NPs

MnO NPs were biosynthesized using MnCl_2_·4H_2_O as a parent compound (precursor) and the fungal extract as reducing, stabilizing, and capping agents. MnO NPs were synthesized by mixing 100 ml of a fungal extract with 100 ml of 1 M MnCl_2_.4H_2_O, subsequently stirred continually at 60 °C for 2 h (Incubation 1). The reaction color changes from yellow to yellowish-brown due to Mn ion reduction. The excess amount of fungal extract (50 ml) was added to the produced MnO NPs and incubated under stirring conditions (200 rpm) at room temperature (28 °C) for 5 h (Incubation 2). Then the reaction color changes from yellowish-brown to reddish-brown due to the capping phase (which can be easily recognized by the naked eye)^6,27^. Finally, the fungus-synthesized MnO NPs were centrifuged at 10,000 rpm for 20 min and washed three times with distilled water and ethanol. Then, the brown residue was dried in an oven at 50 °C for 24 h. A mortar and pestle were used for crushing the MnO NPs to fine powder estimated (g/l) and stored in capping vials. Through the well-diffusion method, phytopathogenic fungi and bacteria estimated the anti-phytopathogenic activity of biosynthesized MnO NPs.

### Instrumental characterization and verification of biosynthesized MnO NPs

UV–Visible absorption spectra of fungus-fabricated MnO NPs were measured on a Shimadzu UV–Vis spectrophotometer (Shimadzu, Tokyo, Japan). FTIR spectra analysis for fungal extracellular fraction and fungus-fabricated MnO NPs were recorded on (Shimadzu FTIR-8400 S, Japan), in the spectral range of 4000–400 cm^−1^. SEM: Morphological structure of MnO NPs was detected by scanning electron microscope (SEM) investigation using a (JEOL JSM 6360LA, Japan) with its EDX combined unit. Thermal stability of fungus-fabricated MnO NPs was determined using the thermogravimetric analysis (TGA) model (TGA/DSC, Shimadzu, Japan), with a heating rate of 20 °C/min under nitrogen flow. X-ray diffraction patterns (XRD) were obtained using (Shimadzu 7000 diffractometer, Japan), operating with *CuK*_*α*_ radiation (*λ* = 0.15406 nm) generated at 30 kV and 30 mA with the scan rate of 2°/min for *2θ* values between 20 and 80 °C. The average crystal size was estimated using the Scherrer’s formula as shown in Eq. (1); Where Y is the fungus-fabricated MnO NPs crystal size, λ is a wavelength of *CuK*_*α*_ radiation, *β* is a full-width half maxima of the different peak and θ is a half diffraction angle.

1 $$Y=(0.9\lambda )/\beta cos\theta .$$

### Molecular identification of mycoendophytic isolate

Among five isolated endophytic fungi, one isolate appeared the high frequency of producing fungus-fabricated MnO NPs, which have proficient anti-phytopathogenic activities, were chosen for further investigations. To determine the ribosomal internal transcribed spacer (ITS), the fungal genome was extracted and purified using *Minipreps* *DNA* *Kit* was obtained from (Promega, USA). ITS region was amplified using PCR with 10 pmol of ITS1 primer (5′-CTTGGTCATTTAGAGGAAGTAA-3′) and 10 pmol of ITS4 primer (5′-TCCTCCGCTTATTGATATGC-3′). This reaction was set up by mixing 100 mg of fungal DNA, 2.5 mM each dNTP, and 1.5 mM MgCl_2_, with 0.4 μl of 500 U Taq DNA polymerase^42^. The initial denaturing temperature was 95 °C was extended to 5 min, and the final extension was extended to 10 min at 72 °C. PCR products were separated by electrophoresis and viewed using a UV trans-illuminator. According to electrophoretic migration, the interested PCR products were eluted from the gel using silica spin columns (DNA Clean & Concentrator, Zymo Research). The purified PCR product was sequenced directly according to the protocol recommended by the manufacturer (Model 3130 automated DNA Sequencer, Genetic Analyzer, Applied Biosystems, Hitachi, Japan). The sequence was compared to the homologous sequences using NCBI BLAST available at http://www.ncbinlmnih.gov/. Multiple sequence alignment was carried out using *BioEdit* software (Hall, 1999), and a phylogenetic tree was constructed using the neighbor-joining (NJ) method through a *MEGA6* program.

### Screening of proficient bioactive metabolites into fungal cellular compartments

The extracted bioactive metabolites were used as reducing, capping, and stabilizing agents in the fabrication of MnO NPs. So, extracellular and cytoplasmic fractions were extracted to detect the proficient fungal cellular compartment that was produced the highest MnO NPs quantities. The comparative evaluation strategy for fungal cultivation media that produced the maximum metabolites was also studied. First, the used growth media was chosen as reported elsewhere^43–45^ as shown in (Table 1S, Supplementary materials). All tested media (50 ml) were prepared in 250 ml flasks and autoclaved separately for 15 min at 121 °C. In the case of wheat bran medium (WBM), wheat bran extract (WBE) was prepared by mixing 10 g of the oven-dried wheat bran with 90 ml distilled water at 60 °C for 5 h, then incubating at 100 °C for 1 h. After that, the extracted nutrients were separated by a filtration system at adjustable pH 7.0^46^. This filtrate was completed to a total volume of 100 ml with distilled water and autoclaved separately for 15 min at 121 °C. All sterilized media were inoculated by fungal inoculums (5%, v/v) and cultivated at 28 °C for 48 h (150 rpm). An ultra-filtration system collected the fungal biomass to prepare extracellular fraction. Cytoplasmic fraction was extracted by suspending the collected fungal biomass in 50 mM PBS (pH 7.0), then sonicated at 40–50% duty for 25–30 bursts. Finally, obtained fractions were centrifuged for 20 min at 10,000 rpm, then stored in clean glass screw bottles at 4 °C. All fractions' total protein and carbohydrate contents were determined using a *Commercial-Rad* colorimetric protein assays kit and carbohydrate colorimetric assay kit (Milpitas, CA 95035 USA). The phytochemical analysis (alkaloids, flavonoids, tannins, phenols, steroids, saponins, and terpenoids contents) were detected in comparative studies to determine the proficient cellular fraction that acted as reducing, capping, and stabilizing agents^21^. Finally, the fabrication of MnO NPs reactions was set up as described previously to use its dried weight in the comparative studies.

### Optimization of fungal biomass production

The used bioprocessing strategy was started with developing effective conditions for extraction of soluble nutrients from wheat bran powder. Since the extraction method affected the amounts of carbohydrates, proteins, amino acids, and other trace elements extracted from wheat bran. So, there are many factors such as Wheat bran powder (50, 100, and 150 g/l), extraction time (3, 12 and 24 h), pH (6, 7, and 8), and extraction temperature (30, 50, and 70 °C), were studied in these experiments. Second, the whole medium ingredients and fungal growth conditions that affected biomass production were optimized. Accordingly, successive statistical optimization approaches (*Plackett–Burman* and *Box–Behnken* designs) were applied to produce the highest fungal cell mass weight. Since the Plackett–Burman design was employed to screen the significant factors that affect fungal biomass production. Subsequently, Box–Behnken factorial design was applied to correlate the relationships between the significant factors and the produced fungal biomass weight.

#### Plackett–Burman design

This experimental design was employed to optimize the fungal biomass production by investigating the effect of different culturing variables. For this study, the used matrix consisted of 12 experiments (trails) and 9 independent variables (inoculums age, agitation, pH, CuSO_4_, incubation temperature, WBE (%), glucose, inoculums size, and MgSO_4_), each of which has a low level (−) and high level (+). All trials were performed in triplicate to detect the significant variables, and the mean values of fungal biomass production (response) were calculated. The final data were analyzed statistically to determine the *p*-value and the confidence levels using standard regression analysis. Subsequently, the first-order polynomial model equation (Eq. 2) was applied by an F-test, where Y is the response, *β*_*o*_ is the model's intercept, *β*_*i*_ is the variable estimate, and *X*_*i*_ is the variable^37,47^. Furthermore, each factor's effect was determined using Eq. (3); where *E*(*x*) is the fungal biomass production, *N* is the number of trails, *M*^+^ and *M*^*-*^ are the effect of this factor at their levels.

2 $${Y}_{Biomass\,production}={\beta }_{0}+\sum {\beta }_{i}{X}_{i}\left(i= 1,2,3,\dots ,K\right),$$

3 $$E\left(x\right)= 2(\sum {M}^{+}-{M}^{-})/N.$$

#### Box–Behnken design

A three-level design is a powerful statistical method used to optimize the microbial fermentation process by examining the interplay of important factors on biomass output. The chosen three independent parameters (pH, WBE, and Inoculum size) were tested at three levels, coded −, 0, + for low, middle, and high values, respectively, to compute and estimate the ideal point for maximal biomass production. The response variable was fitted via a second-order polynomial model (Eq. 4) to correlate the dependent response to independent variables using *Minitab* *18.1* software. The quality of the fit of the polynomial model equation was expressed by the coefficient of R^2^^39,40^. Actual values of each factor were determined by using *Myers* and *Montgomery* equation (Eq. 5); where, *Y* is predicted response, *β*_*o*_ is the intercept, *β*_*i*_ is the linear coefficient, *β*_*ij*_ is the quadratic coefficient, β_ii_ is linear-by-linear interaction between *X*_*i*_ and *X*_*j*_ is regression coefficients, and *X*_*i*_*X*_*j*_ are input variables that influence the response variable Y^37^.

4 $$Y= {\beta }_{0}+ \sum_{i=1}^{K}{\beta }_{i}{X}_{i}+\sum_{i=1}^{K}{\beta }_{ii}{X}_{ii}^{2}+\sum_{i=1, i<j}^{K}\times \sum_{j=2}^{K}{\beta }_{ij}{X}_{i}{X}_{j}+\varepsilon ,$$

5 $$Coded\; value=Actual\; value-\left(\frac{(High\; Level+Low \; Level)/2}{(High \; Level-Low \; Level)/2}\right).$$

### Industrial biotechnological strategies of scaling-up production of biosynthesized MnO NPs

Tested fungal strain was inoculated firstly on potato dextrose agar plates and incubated at 30 °C under static conditions for 14 days. Then, the spore suspension was prepared by a sterile saline solution (0.9%) under aseptic conditions. These fungal spores were counted via hemocytometer to prepare suitable spore suspension for inoculating step. The secondary cultivation was prepared by inoculating 500 ml of statistically optimized medium in shaken bottles (1000 ml) with 0.5 ml of freshly prepared fungal spore suspension (3 × 10^4^ spores/ml) and incubated at 30 °C. Finally, 7 L BioFlo 310 bioreactor (New Brunswick Scientific, Edison, NJ, USA), contained 4.5 L of the sterile medium was inoculated, then the temperature and pH were controlled at 30 °C and 5.5, respectively. The mixing characteristic of aeration/agitation rates and timing were studied using fungal biomass and MnO NPs production as responses via seven control strategies in batch fermentation mode. The tested strategies were designed as described in Table 10. During the fermentation periods, the samples were taken to calculate the biomass and MnO NPs production.

**Table 10:** Optimization of the fungal biomass and MnO NPs production by studying different aeration/agitation rates and its timing, all the way via controlled batch fermentation modeusing7L bioreactor.

Batch runs	Airflow (vvm)	Agitation speed (RPM)
B1	1–2.5 vvm/12 h	100–800 rpm/6 h
B2	0.1–0.5 vvm/12 h	100–800 rpm/3 h
B3	0.5–3.5 vvm/12 h	50–250 rpm/6 h
B4	0.1–4.5 vvm/6 h	400 rpm
B5	2 vvm	50–300 rpm/6 h
B6	Airflow and agitation speeds were adjusted to let the dissolved O_2_ level did not below 10%	
B7	Airflow and agitation speeds were adjusted to let the dissolved O_2_ level did not below 40%	

The endpoint of the fermentation period was detected by increasing the concentration of dissolved oxygen (DO), indicating the carbon source's depletion^48,49^. So, at this point, the four feeding strategies were tested using different fed-batch fermentation modes. Tested four fed-batch strategies were set up by whole optimized medium ingredients or WBE only as described in Table 11. The airflow rate and agitation speed were controlled to let DO level did not below 10% at all tested fed-batch fermentation modes. In the log phase, fungal cell mass increases with time exponentially. While the behavior of fungal growth and produced MnO NPs were described and determined kinetically via different fermentation models using different equations^30,32^. The yield coefficient was determined by consumed carbon source and yielded biomass. So, many parameters such as biomass yield coefficient (***Y***_***X/S***_), maximum biomass (X_max_), maximum MnO NPs yield (P_max_), and maximum specific growth rate (µ_max_) were calculated.

**Table 11:** Optimization of the fungal biomass and MnO NPs production by studying different feeding strategies**,** feeding regimesandfeeding volumes through controlled fed-batch fermentation modeusing7L bioreactor.

Fed-batch runs	Feeding strategies	Feeding regimes	Feeding volumes
FB1	Whole medium ingredients	Constantly	4 ml/6 h
FB2		Exponentially via pulsed feeding	1.0–16.0 ml/6 h
FB3	Wheat bran extract only	Constantly	10 ml/6 h
FB4		Exponentially via pulsed feeding	2.0–40.0 ml/6 h

### Statistical optimization strategy for biosynthesis reaction of MnO NPs

Biosynthesis reaction of MnO NPs was affected by different ingredients and reaction conditions such as precursor concentration (parent metal), fungal extract concentration (reducing/capping agents), pH, incubation temperature, incubation time (reaction time), and stabilizing agent (fungal extract). Taguchi design was applied to maximize MnO NPs production via this optimization strategy's proficient biological fabrication steps. So, in these experiments, L_25_ Taguchi orthogonal array design (5**6) was selected to perform this optimization strategy, while MINITAB 18 software was used and Eqs. (6–9), where fungus-fabricated MnO NPs mass weight was determined, and maximum signal to noise ratios (S/N) were calculated for each fabrication reaction^41^. Finally, the validation test was conducted based on *Taguchi’s* results, and the predicted S/N ratios were calculated.

6 $${{Y}^{-}}_{(meanresponse)}=\frac{1}{n}\sum_{i=1}^{n}{Y}_{i},$$

7 $${S}_{(standarddeviation)}=\sqrt{\sum_{i=1}^{n}\frac{{({Y}_{i}-{Y}^{-})}^{2}}{n-1}},$$

8 $$S/N_{(Larger)}= -10Log\left(\left(\frac{1}{n}\right)\sum_{i=1}^{n}\frac{1}{{Y}_{i}^{2}}\right),$$

9 $$S/N_{(Predicted)}=\left(\frac{S}{{N}_{m}}+\sum_{i=1}^{n}\left|\frac{S}{{Y}^{-}}-\frac{S}{{N}_{m}}\right|\right).$$

### Antagonistic potential for biosynthesized MnO NPs against some phytopathogens in-vitro

The minimum inhibitory concentration (MIC) of fungus-fabricated MnO NPs was recorded and calculated. Also, antagonistic activities of fungus-fabricated MnO NPs against phytopathogens (bacteria and fungi) were observed and determined via a well-diffusion method^23^. Since phytopathogenic fungi were cultivated in Czapek medium, that contained (g/l): NaNO_3_, 3.0; K_2_HPO_4_, 1.0; MgSO_4_∙7H_2_O, 0.5; KCl, 0.5; FeSO_4_∙7H_2_O, 0.01; and agar, 15 (pH 4.8). In addition, phytopathogenic bacteria were inoculated into nutrient agar medium composed of (g/l): [peptone 5, sodium chloride 5, beef extract 1.5, yeast extract 1.5, and agar 15 (pH 7.0)]. Different concentrations of fungus-fabricated MnO NPs (10, 50, 90, 130, 170, 210, 250, 290, 330 and 370 µg/ml) were prepared and loaded into wells (0.5 mm). Then, these plates were incubated at 30 °C for 24–72 h and examined for inhibition zones. Finally, maximum bactericidal concentration (MBC) and maximum fungicidal concentration (MFC) were checked and determined^50^. These experiments were conducted in triplicates, and these data were statistically analyzed via the one-way *ANOVA* using *Minitab* software.

## Supplementary Information

Supplementary Information.

## Data Availability

The article includes the data used to support the results of a study. Authors clarify that no further approvals were necessary to conduct research on plant material, in accordance with local and institutional guidelines. This isolate was submitted in the GenBank database (https://www.ncbi.nlm.nih.gov/nuccore/MF429775) as endophytic *Trichoderma* *virens* strain EG92 with the accession number MF429775 because it was completely similar to *Trichoderma* *virens.*
