# Supplementary material for: Novel biosynthesis of MnO NPs using Mycoendophyte: industrial bioprocessing strategies and scaling-up production with its evaluation as anti-phytopathogenic agents
Source: Sci Rep. 2023 Feb 4;13:2052. doi: 10.1038/s41598-023-28749-z (PMC9899258; doi:10.1038/s41598-023-28749-z)
Supplement: Supplementary file 1 — Supplementary Information. [file 41598_2023_28749_MOESM1_ESM.docx]

**Supplementary Materials**

**Table (1S):** Tested broth media that used for fungal growth evaluation, Czapek-Dox medium (CDM), Yeast Malt medium (YMM), Synthetic medium (SM), Glucose Soya bean meal medium (GSM), Yeast glucose medium (YGM), and Wheat bran medium (WBM).

| **Medium Codes** | **CDM** | **YMM** | **SM** | **GSM** | **YGM** | **WBM** |
| --- | --- | --- | --- | --- | --- | --- |
| ***Medium ingredients***  ***(g/L)*** | Sucrose, 30.0 | Malt extract, 6.0 | (NH_4_) H_2_PO_4_, 2.0 | Glucose, 10.0 | Yeast extract, 4.0 | Wheat bran extract (WBE) 20% (v/v) |
|  | (NH_4_)_2_SO_4_, 2.0 | Maltose, 1.8 | Dextrose, 20.0 | Soybean meal, 10.0 | Glucose 20 | Glucose, 5.0 |
|  | NaNO_3_, 1.0 | Dextrose, 6.0 | K_2_HPO_4_, 1.0 | NaCl, 10.0 | K_2_HPO_4_, 20 | KH_2_PO_4_, 1.0 |
|  | K_2_HPO_4_, 1.0 | Yeast extract, 1.2 | MgSO_4_·7H_2_O, 0.5 | CaCO_3_, 1.0 | MgSO_4_·7H_2_O 0.5 | MgSO_4_·7H_2_O, 0.5 |
|  | KCl, 0.5 |  | CaCl_2_·2H_2_O, 0.04 |  |  | CuSO_4_ 0.1 |
|  | MgSO_4_·7H_2_O 0.5 |  | FeSO_4_·7H_2_O, 0.005 |  |  |  |
|  | FeSO_4_.7H_2_O, 0.01 |  | ZnSO_4_·7H_2_O, 0.0005 |  |  |  |
